# Supplementary material for: Self-perception of physical appearance of adolescents and associated factors in Addis Ababa, Ethiopia
Source: PLoS One. 2023 Jan 27;18(1):e0281202. doi: 10.1371/journal.pone.0281202 (PMC9882736; doi:10.1371/journal.pone.0281202)
Supplement: Attachment — Submitted filename: Comments for Manuscript ID PONE-D-22-00860.pdf [file pone.0281202.s004.pdf]

PLOS ONE

Self-perception of physical appearance of adolescents and associated factors in Addis Ababa, Ethiopia

--Manuscript Draft--

**Manuscript Number:**	PONE-D-22-00860R1
**Article Type:**	Research Article
**Full Title:**	Self-perception of physical appearance of adolescents and associated factors in Addis Ababa, Ethiopia
**Short Title:**	Perception of physical appearance of adolescents
**Corresponding Author:**	ZIYAD AHMED ABDO, Master<br>Ethiopian Public Health Association<br>Addis Ababa, Addis Ababa ETHIOPIA
**Keywords:**	self-perception; Adolescent; Physical Appearance; ADDIS ABABA; Ethiopia
**Abstract:**	Introduction<br>Establishing a positive body image is a critical factor for adolescents' physical and mental health, as it determines self-confidence, sustainable individual growth and development throughout their lives. This reality needs to be supported by evidences generated locally. However, there is a lack of information in this regard in the study setting. Thus, the aim of study was to generate evidence by assessing self-perception of physical appearance and its associated factors among adolescents in Addis Ababa.<br><br>Methods and materials<br>A community-based cross-sectional study design was used to conduct the study. At the end of the multistage sampling procedure, a systematic random sampling technique was employed to select 308 study participants from selected districts. The questionnaire was adapted from previous studies as it was appropriate for local context. The data collectors were trained before the data collection and supervised during the data collection period, and the questionnaire was pretested. Logistic regression was employed to identify the predictor variables. Statistical significance was considered at P <0.05 with adjusted odds ratios calculated at 95% CIs.<br>Result<br>The overall good self-perception of one's physical appearance was 48.4% [95% CI=48.8, 541]. Having BMI between 18.5 kg/m 2 -24.9 kg/m 2 (AOR=2.56; 95% CI: 1.45, 4.54), presence of enough sport fields in the school (AOR=1.89; 95% CI: 1.09, 3.29), having daily access to internet services (AOR=1.69; 95% CI: 1.07, 2.94), following Ethiopian movies/cinemas (AOR=2.46; 95% CI: 1.46, 4.15), and regularly following western movies/cinemas (AOR=2.0; 95% CI: 1.11, 3.59) were significantly associated with good self-perception of one's physical appearance.<br>Conclusion<br>According to this study, good self-perception of one's physical appearance was low. Adolescents and their family need to work to bring normal body weight and height, which in turn brings their normal body mass index, which in turn will increase good self-perceptions of their physical appearance. Schools and the local administrations need to increase access to enough sport fields for students to strengthen their physical fitness, which increases their good self-perception of their physical appearance.
**Order of Authors:**	ZIYAD AHMED ABDO, Master
	Semira Ahmed Seid
	Aynye Negesse
**Response to Reviewers:**	Dear Editor,<br>Thank you for giving us the opportunity to submit a revised draft of our manuscript titled (Self-perception of physical appearance of adolescents and associated factors in Addis Ababa, Ethiopia) to Plos One journal. We appreciate you and the reviewer for your precious time in reviewing our paper and providing valuable suggestion. It was your valuable and insightful comments that led to possible improvements in the current version. The authors have carefully considered the comments and tried our best to

address every one of them. We hope the manuscript after careful revisions meet your high standards. The authors welcome further constructive comments if any.
Below we have provide the point-by-point responses/clarification to editor and reviewer comments. In addition, all modifications in the manuscript have been highlighted in red in document uploaded named as “Revised Manuscript with Track Changes”.
1.Comments from Editor:
Comment 1: Please ensure that your manuscript meets PLOS ONE's style requirements, including those for file naming.
Response: We had revised the manuscript as per PLOS ONE’s style requirements including the file naming.
Comment 2: Statement related to financial disclosure.
Response: The authors received no specific funding for this work. The same statement is included in the manuscript.
Comment 3: Issue related to data availability.
Response: We provide the baseline data on protocols.io (https://doi.org/10.17504/protocols.io.8epv5j635l1b/v1)
Comment 4: Your ethics statement should only appear in the Methods section of your manuscript.
Response: The ethical statement is included under method section by removing from declaration part.
Comment 5: Thank you for submitting the above manuscript to PLOS ONE. During our internal evaluation of the manuscript, we found significant text overlap between your submission and the following previously published works, some of which you are an author.
Response: We have made great effort to replace, Re-write and adjust those overlapping text.
2.Comments from the reviewer:
Abstract section comments:
Comment 1: Introduction: The clear research gap is not clearly stated. The researcher describes the importance of positive body image for physical and mental health. However, the research what is unknown and what the current research adds a new knowledge are not well described.
I would suggest remove the highlighted words from the aim of the study “Therefore, the aim of this study was to assess the level of self-perception of physical appearance and its associated factors among adolescents in Addis Ababa, Ethiopia.”
Response: Thank you for your valuable comment. We have tried to show the research gap and what the current research add to current knowledge. Moreover, as abstract word count is limited it is impossible to explain more in this part, so that we had tried to explained more in the introduction part.

Comment 2: The researcher did not describe how the multistage sampling was done. Its needs a summary of report for the multistage sampling in abstract section because the reader does not understand the sampling units were schools, or districts, or others. It is also better to describe what variables are measured and how before they tell us the analysis. For example, self-perception is measured using structured questionnaire (it is good to mention whether it is validated tool or tools were developed for this study) and describe how the data was collected such as using ‘self-administered’ or ‘interview’. Some other detail information can be presented in the methods section of the manuscript. I also suggest to define the self-perception classification if it has. For example, the researcher said the “over all good self-perception”, what does it mean? The researcher also reported there was also said the good self-perception was low in the conclusion section which need definition in the method section. Indeed, summary of the important methods section needs further improvement.
Response: Thank you again for the comment: The summary of report for the multistage sampling is included in the abstract part as per your comment.

The variables measured are included in the manuscript under method section subsection study variables.

The dependent variable are defined under methodology section

We had tried to modify the methodology part to make it clearer to the reader by incorporating all your comments. Please see the methodology section of the manuscript. As you know, under abstract section it is difficult to explain every points as this part word count allowed is very limited as per the journal format.
Comment 3: Having BMI between 18.5 kg/m2-24.9 kg/m2 (AOR=2.56; 95% CI: 1.45,

Powered by Editorial Manager® and ProduXion Manager® from Aries Systems Corporation

4.54), presence of enough sport fields in the school (AOR=1.89; 95% CI: 1.09, 3.29), having daily access to internet services (AOR=1.69; 95% CI: 1.07, 2.94), following Ethiopian movies/cinemas (AOR=2.46; 95% CI: 1.46, 4.15), and regularly following Western movies/cinemas (AOR=2.0; 95% CI: 1.11, 3.59) were significantly associated with self-perception of one's physical appearance

Does it associated with …good self-perception low self-perception? Make it clear or you may say "had statistically significant positive association with self-perception of one's appearance

Response: The comment is well received and corrected accordingly in the manuscript.

Comment 4: Conclusions: the researcher need to know the nature of a cross-sectional study to determine the associated factors. Thus, researcher must be curious on the factors associated with self-perception which does not mean a causes. There might be various factors that may also associated with self-perception, for example the economy, the social factors, psychological factors, and other factors. The researcher made a shallow recommendation on how the current knowledge is used.

The researcher recommends to increase the access to sport fields …not clear. This look a very general recommendation one can do without

Key words ….>keywords

Response: Of course there might be many factors that associated with the self-perception of one's physical appearance other than that discussed here. However, we explained and discussed the factors depending the result of our analysis. In addition, as you know the recommendation must be depend on the finding of the study. Anyhow we have adjusted the recommendation to some extent.

It is not clear about comment of "Key words" We have included the possible keywords in the manuscript.

Introduction section comments

Comment 5: It would be good to include a paragraph explaining how self-perception may be influenced by a variety of factors.

It is inconsistent throughout the document to use terms like 'dissatisfaction' and 'low self-perception'.

Responses: We have included a paragraph explaining how varies factors do affect self-perception of physical appearance.

In relation to 'dissatisfaction' and 'low self-perception', some literatures use the first, other use the second one. Hence, in the introduction section part of this manuscript, there may be either of them or both because more of the introduction part was extracted from previous studies that might use either of them to explain all most the same issue. However, the finding of this study is depend on the operational definition we had provided in the methodology part which is either Good self-perception or Poor self-perception.

Comment 6: The study was a community based cross sectional study. Why the study include only adolescent who attend high school education? Would not be easy to make institution (based) cross-sectional study if the participants are only adolescent who attend high school education?

Response: To make this point clear, this study was first planned to be done at institutional (school) level, however, due to covid-19, the schools were closed as part of covid-19 prevention protocol. Then we have changed to community based. So, the sampling unit is household not school.

Comment 7: The researcher selects two sub-cities from Addis Ababa, Ethiopia from 10 or now is 11 sub-cities. Do you think that the result of the study generalizable to Addis Ababa?

Response: At the time of starting of this work, the sub cities in Addis Ababa were 10. Hence, we have selected only two sub cities due to limitation of available resources. However, as much as possible, we tried to make the sampling representative by using a simple random sampling technique to select the sub cities and districts.

Comment 8: I also highly recommend a thorough formatting of the paper for publication in the journal.

Response: We have tried to format the paper according to the journal formatting style.

Comment 9: The researcher did not mention how the two sub-cities selected? Was it randomly or purposively?

Response: The two sub cities was selected by simple random sampling system.

Comment 10: Is the sampling unit was household or school? This is not clear for the reader

Response: The sampling unit was household. This study was first planned to be done at institutional (school) level, however, due to covid-19, the schools were closed as part

Powered by Editorial Manager® and ProduXion Manager® from Aries Systems Corporation

	of covid-19 prevention protocol. Then we have changed to community based. So, the sampling unit is household not school.<br>Comment 11: How many household or school was included in the study?<br>Response: 308 households was included.<br>Comment 12: It is also not clear how simple random sampling was applied at community based cross-sectional study or was it school based<br>Response: The comment is well taken. It is typing error. Corrected to Systematic random sampling system.<br>Comment 13: The sample size calculation not clearly described. What was the proportion of 19.5%? Was it dissatisfaction on self-perception? Why the researcher uses the proportion 50% which can give the maximum sample size for the cross-sectional study. The researcher also did not describe the adjustment of sample size because of multistage sampling strategy.<br>Response. Corrected and we had included sample size calculation description. 19.5% is the proportion of satisfaction not dissatisfaction. As you know 50% is recommended if there is no any previous study done on the topic. Thus why we used the available reference.<br>Comment 13: Dependent Variable: Level of self-perception of physical appearance: bring the operational definition here.<br>Response: We had already included operational definition in methodology part. However, as per your comment we have merged with dependent variable.<br>Comment 14: The researcher is not consistently used terms in relation to self-perception. In the introduction section they used terms 'dissatisfaction' and in the method they used 'good-self-perception' and 'low-self-perception'.<br>Response: In relation to 'dissatisfaction' and 'low self-perception', some literatures use the first, other use the second one. Hence, in the introduction section part of this manuscript, there may be either of them or both because more of the introduction part was extracted from previous studies that might use either of them to explain all most the same issue. However, the finding of this study is depend on the operational definition we had provided in operational definition in the methodology part which is either Good self-perception or Poor self-perception.<br>Comment 15: Why the researcher wants to use the mean score to classify the self-perception as mean can be affected by skewness. Did the researcher checked the normal distribution of the data before they decide to use the mean score classification?<br>Responses: We used mean as cut-point, because our data is normally distributed data.<br>Comment 16: The classification term also makes confuse the reader, for example, in the abstract section the researchers said, "Good perception of one's physical appearance was low". This makes confused with the self-perception classification ''Good" and "low". I would suggest "good" and "poor' or "high" and "low" or "satisfied" and "dissatisfied"<br>Responses: It good and appropriate comment, so, as per your comment we corrected the operational definition as high" and "poor" satisfaction. As such, this operational definition was used to describe the satisfaction level of the finding of the study.<br>Comment 17: I would suggest making the operational definition together with the dependent variable.<br>Response: Comment total accepted, the operational definition merged with dependent variable.<br>Comment 18: The researcher did not clearly describe the variables which needs revision<br>Response: Comment accepted and revised accordingly.<br>Comment 19: The researchers did not clearly describe the measurements in details. For examples to measure the self-perception of physical how many questions were used and on what type? Was it liker or yes/No answer options.<br>Response: Comment total accepted and corrected accordingly. See Methodology part sub section Data collection tools and quality control which explain the measurments.<br>Comment 20: There is a repetition of statement<br>Prior to the actual data collection, the questionnaire was adjusted and 18 corrected based on the pretest result, and the final questionnaire was translated to Amharic and 19 then back to English to ensure consistency.<br>"The questionnaire was pretested and modified 25 based on the findings related to clarity, wordings, logical sequence, skip patterns of the questions 26 and resources needed."<br>Merge these two statements into one

Powered by Editorial Manager® and ProduXion Manager® from Aries Systems Corporation

	Response: Comment Accepted. Merged accordingly.<br>Comment 21: The researcher did not explained why BMI and MUAC measurement were used together? Study showed BMI and MUAC highly correlated in Adolescent. What was the assumptions behind using this two indices for nutritional assessment?<br>Responses: The comment is right, different literatures showed that BMI and MUAC measurements are highly correlated. We assumed that the findings of our study may show the research communities another support in addition to available evidences if both measurements are done simultaneously. Besides, the assumption behind using these two indices for nutritional assessment among adolescents proving researchers thoughts such as "The MUAC is useful especially in monitoring severe under nutrition" and "The BMI is a commonly used measurement for screening both underweight and overweight".<br>Comment 22: Were the researchers considering any assumptions for the appropriateness of the analysis they used? Why logistics regression analysis? Did the researcher check the assumption for it?<br>Response: As our data is categorical data, we used logistics regression analysis which does not require linearity test. We have made same assumptions before starting the analysis. Example we have checked multi-collinearity test and our data is free from it.<br>Comment 23: According to WHO BMI-for age (Obese: >2SD; Overweight: >1SD; Normal: 1 - -2SD; Thinness: <−2 SD; Severe thinness: <−3 SD) and height-for-age below -2SD is used for stunting. The MUAC for age can also be used classified for adolescent age. The researcher did not clearly describe the BMI and MUAC classification in this regard. This needs to be clearly stated with the reference. This need to be clearly stated with the reference.<br>Response: We included the BMI classification explanation with reference in the methodology part according to your comment. Please see sub section data collection tools and quality control under methodology part.<br>Result section comments<br>Comment 24: What was the base for monthly income classification?<br>Responses: The classification of income is depend on the mean value of the income level taken as cut-off point.<br>Comment 25: According to responses rated based on five Likert scales, namely, 1-strongly disagree, 2- 4 disagree, 3-not sure, 4-agree and 5-strongly agree, the overall mean perception of respondents 5 was 2.9 (±0.02). Based on the definition of good and low perception, approximately 157 (55.5%) 6 respondents had good perception, and 126 (44.6%) had low perception.<br>The above statement is not clear for reader<br>Response: To make it more clear, we re-write it and add a table
**Additional Information:**	
**Question**	**Response**
**Financial Disclosure**<br><br>Enter a financial disclosure statement that describes the sources of funding for the work included in this submission. Review the submission guidelines for detailed requirements. View published research articles from *PLOS ONE* for specific examples.<br><br>This statement is required for submission and **will appear in the published article** if the submission is accepted. Please make sure it is accurate.	No

**Unfunded studies**<br>Enter: *The author(s) received no specific funding for this work.*<br><br>**Funded studies**<br>Enter a statement with the following details:<br>• Initials of the authors who received each award<br>• Grant numbers awarded to each author<br>• The full name of each funder<br>• URL of each funder website<br>• Did the sponsors or funders play any role in the study design, data collection and analysis, decision to publish, or preparation of the manuscript?<br>• **NO** - Include this sentence at the end of your statement: *The funders had no role in study design, data collection and analysis, decision to publish, or preparation of the manuscript.*<br>• **YES** - Specify the role(s) played.<br><br>* typeset	
**Competing Interests**<br><br>Use the instructions below to enter a competing interest statement for this submission. On behalf of all authors, disclose any competing interests that could be perceived to bias this work—acknowledging all financial support and any other relevant financial or non-financial competing interests.<br><br>This statement is required for submission and **will appear in the published article** if the submission is accepted. Please make sure it is accurate and that any funding sources listed in your Funding Information later in the submission form are also declared in your Financial Disclosure statement.<br><br>View published research articles from *PLOS ONE* for specific examples.	The authors have declared that no competing interests exist.

**NO authors have competing interests**<br><br>Enter: *The authors have declared that no competing interests exist.*<br><br>**Authors with competing interests**<br><br>Enter competing interest details beginning with this statement:<br><br>*I have read the journal's policy and the authors of this manuscript have the following competing interests: [insert competing interests here]*<br><br>* typeset	
**Ethics Statement**<br><br>Enter an ethics statement for this submission. This statement is required if the study involved:<br><br>• Human participants<br>• Human specimens or tissue<br>• Vertebrate animals or cephalopods<br>• Vertebrate embryos or tissues<br>• Field research<br><br>Write "N/A" if the submission does not require an ethics statement.<br><br>General guidance is provided below. Consult the submission guidelines for detailed instructions. **Make sure that all information entered here is included in the Methods section of the manuscript.**	Before conducting the study, ethical clearance was obtained from the Addis Ababa Health Bureau ethical clearance board and Addis Ababa educational bureau. Participants' right to self-determination and autonomy was respected, and study participants were given any information they needed verbally and in written prior to self-administration. For those who were below 18 years old, consent from their parents was obtained. The rights of each respondent refusing to answer a few or all questions were respected. Participation was voluntary, and participants could withdraw from the study at any time without explanation

**Format for specific study types**<br><br>**Human Subject Research (involving human participants and/or tissue)**<br>• Give the name of the institutional review board or ethics committee that approved the study<br>• Include the approval number and/or a statement indicating approval of this research<br>• Indicate the form of consent obtained (written/oral) or the reason that consent was not obtained (e.g. the data were analyzed anonymously)<br><br>**Animal Research (involving vertebrate animals, embryos or tissues)**<br>• Provide the name of the Institutional Animal Care and Use Committee (IACUC) or other relevant ethics board that reviewed the study protocol, and indicate whether they approved this research or granted a formal waiver of ethical approval<br>• Include an approval number if one was obtained<br>• If the study involved *non-human primates*, add *additional details* about animal welfare and steps taken to ameliorate suffering<br>• If anesthesia, euthanasia, or any kind of animal sacrifice is part of the study, include briefly which substances and/or methods were applied<br><br>**Field Research**<br><br>Include the following details if this study involves the collection of plant, animal, or other materials from a natural setting:<br>• Field permit number<br>• Name of the institution or relevant body that granted permission	
**Data Availability**<br><br>Authors are required to make all data underlying the findings described fully available, without restriction, and from the time of publication. PLOS allows rare exceptions to address legal and ethical concerns. See the PLOS Data Policy and FAQ for detailed information.	Yes - all data are fully available without restriction

Powered by Editorial Manager® and ProduXion Manager® from Aries Systems Corporation

A Data Availability Statement describing where the data can be found is required at submission. Your answers to this question constitute the Data Availability Statement and **will be published in the article**, if accepted.<br><br>**Important:** Stating 'data available on request from the author' is not sufficient. If your data are only available upon request, select 'No' for the first question and explain your exceptional situation in the text box.<br><br>Do the authors confirm that all data underlying the findings described in their manuscript are fully available without restriction?	
**Describe where the data may be found in full sentences. If you are copying our sample text, replace any instances of XXX with the appropriate details.**<br><br>• If the data are **held or will be held in a public repository**, include URLs, accession numbers or DOIs. If this information will only be available after acceptance, indicate this by ticking the box below. For example: *All XXX files are available from the XXX database (accession number(s) XXX, XXX.)*.<br>• If the data are all contained **within the manuscript and/or Supporting Information files**, enter the following: *All relevant data are within the manuscript and its Supporting Information files.*<br>• If neither of these applies but you are able to provide **details of access elsewhere**, with or without limitations, please do so. For example:<br><br>*Data cannot be shared publicly because of [XXX]. Data are available from the XXX Institutional Data Access / Ethics Committee (contact via XXX) for researchers who meet the criteria for access to confidential data.*<br><br>*The data underlying the results presented in the study are available from (include the name of the third party*	The datasets used and/or analyzed during the current study is uploaded as Supporting Information file.

Powered by Editorial Manager® and ProduXion Manager® from Aries Systems Corporation

and contact information or URL).<br>• This text is appropriate if the data are owned by a third party and authors do not have permission to share the data.<br><br>* typeset	
Additional data availability information:	

Manuscript

Click here to access/download;Manuscript;Manuscript .docx

Self-perception of Physical Appearance of Adolescents and Associated Factors in Addis Ababa, Ethiopia

Ziyad Ahmed Abdo[1*], Semira Ahmed[2], Aynye Negese[3]

[1]Ethiopian Ministry of Health, Department of Hygiene and Environmental Health, Addis Ababa, Ethiopia.

[2]Ethiopian Ministry of Health, Department of Public Relation and Communication, Addis Ababa, Ethiopia.

[3]Ethiopian Ministry of Health, Department of Public Relation and Communication, Addis Ababa, Ethiopia.

Corresponding author: Ziyad Ahmed Abdo, Email: *ziyadahm1982@gmail.com*

Abstract

Introduction

Establishing a positive body image is a critical factor for adolescents' physical and mental health, as it determines self-confidence, sustainable individual growth and development throughout their lives. This reality needs to be supported by evidences generated locally. However, there is a lack of information in this regard in the study setting. Thus, the aim of study was to generate evidence by assessing self-perception of physical appearance and its associated factors among adolescents in Addis Ababa.

Methods and materials

A community-based cross-sectional study design was used to conduct the study. At the end of the multistage sampling procedure, a systematic random sampling technique was employed to

select 308 study participants from selected districts. The questionnaire was adapted from previous studies as it was appropriate for local context. The data collectors were trained before the data collection and supervised during the data collection period, and the questionnaire was pretested. Logistic regression was employed to identify the predictor variables. Statistical significance was considered at P <0.05 with adjusted odds ratios calculated at 95% CIs.

Result

The overall good self-perception of one's physical appearance was 48.4% [95% CI=48.8, 541]. Having BMI between 18.5 kg/m^2-24.9 kg/m^2 (AOR=2.56; 95% CI: 1.45, 4.54), presence of enough sport fields in the school (AOR=1.89; 95% CI: 1.09, 3.29), having daily access to internet services (AOR=1.69; 95% CI: 1.07, 2.94), following Ethiopian movies/cinemas (AOR=2.46; 95% CI: 1.46, 4.15), and regularly following western movies/cinemas (AOR=2.0; 95% CI: 1.11, 3.59) were significantly associated with good self-perception of one's physical appearance.

Conclusion

According to this study, good self-perception of one's physical appearance was low. Adolescents and their family need to work to bring normal body weight and height, which in turn brings their normal body mass index, which in turn will increase good self-perceptions of their physical appearance. Schools and the local administrations need to increase access to enough sport fields for students to strengthen their physical fitness, which increases their good self-perception of their physical appearance.

Key Words: Self-perception, Adolescent, Physical Appearance, Addis Ababa, Ethiopia

Introduction

The problem of body image, self-perception and dissatisfaction with one's appearance is widely discussed in the anthropological and medical literatures (1–4). Physical care and body image, especially during adolescence, are social issues (5). Harter (1999) describes self-perception as to evaluative judgments of attributes within discrete domains such as physical appearance, cognitive competence, athletic competence, social acceptance and so forth (6,7). Thus, self-perception develops through experience in social and physical environment, interactions with significant others, and individual behavioral traits (8,9). As children develop a sense of themselves, interact with the world, and gain experiences, their self-concept is affected. is defined (10).

The physical changes that the body undergoes during adolescence have a profound impact on an individual's personal and social identity (11). For this reason, the process of forming physical self-perception can be considered inherent to this age and can also influence physical exercise habits (5,12). From the above, it is possible to conclude that a good body image can be associated with better self-perception, as well as a more active and less sedentary lifestyle (1). These interpretations are also influenced by significant others' evaluations, reinforcements, and attributions of the individual's behavior (11,13).

School is an important level of education in which adolescents continue to learn the minimum level of knowledge and skills that every citizen should have (9,14). Entering to high school is a transition to a new educational level and can be stressful for adolescents since they encounter a new curriculum, possibly new and different friends, new teachers, preparation for an important and life-changing exam, and entering puberty (14). Specifically, at the secondary level of

education, adolescents acquire problem-solving skills and develop them according to social values and basic skills for functioning in society(3). In addition, this educational process plays a crucial role in the development of self-perception of their physical appearance in adolescents.

Studies have shown that the prevalence of body image dissatisfaction in developed countries ranges from 35% to 81% in adolescent females and 16% to 55% in adolescent males (9,15–17). Despite data-based evidence from developed countries, little is known about the prevalence of adolescent body image and related factors of dissatisfaction in developing countries such as Ethiopia (23).

Several lines of evidence suggest that biological factors such as age, sex, puberty, body composition, and psychological factors such as depression, low self-esteem, and adoption of weight loss strategies are factors for adolescent body image dissatisfaction (17). parents also affect adolescent self-perception through emotion, motivation, beliefs, values, expectancies, and behaviors (18). A study also show that, self-perception of one’s body, weight was the most important characteristics in adolescent (19). Peers are central to children’s psychological and self-image development and (18).

Adolescents face many challenges, such as solidifying their personality, accepting physical changes, leaving their families, building norms and moral values, choosing a career, and becoming a contributing member of society (17). These serious developmental challenges and potential sources of stress can be detrimental to the development of healthy self-perception and positive body image (24). High self-perception and a positive body image have a positive impact on an individual's quality of life, performance, and relationships. Adolescents who fail to

develop a positive body image and self-perception are at risk of depression, other health condition and related personal problems (25).

Although the level of satisfaction with the self-perception of body image of adolescents is a reason for the growing attention of national and international organizations related to education and health promotion, governments, and researchers (19), there are a limited number of studies related to the self-perception of the physical appearance of adolescents, and little is known about the factors related to satisfaction with one's body image. Similarly, to our knowledge, there is no study done on this issue in Ethiopia, specifically in Addis Ababa. As such, this study intends to provide evidence by assessing the magnitude of self-perception of physical appearance and its associated factors among adolescents in Addis Ababa, Ethiopia.

Materials and Methods

Study setting and design

A community-based cross-sectional study design was used to conduct the study. The study was conducted in Addis Ababa, Ethiopia. Addis Ababa is the capital city and largest city of Ethiopia and also the country's political, economic, cultural and tourism center. It is located in the central part of Ethiopia and close to the equator. Sitting at the foot of mount Entoto at an elevation of 2,355 meter. It cover an area of 527 km^2 and has a population size of about 4 million (20).

Population and eligibility criteria

The source population of this study was all adolescents in Addis Ababa city who was attending high school education. The study population was all adolescents in selected districts and

attending high school in selected sub cities. All selected adolescent students in selected districts who were in high school education during the data collection time and in the adolescent age group (10-19 years) were included in the study. Adolescents with chronic health problems, especially those with diabe[illegible] were excluded from the study.

Sample size determination

The required sample size for this study was calculated by using a single population proportion formula with the following assumptions: 95% confidence level, 5% margin of error and 19.5% proportion of satisfaction with one's physical appearance taken from a previous study performed in Brazil (9).

$$n = \frac{(1.96)^2\ 0.195(1-0.195)}{(0.05)^2} = 280$$

By considering 10% non-response rate, a total of 308 sample populations was included.

Sampling techniques

Multistage sampling techniques were used to select study participants. First Kolfe Keranio and Yeka sub city was selected from sub-cities in Addis Ababa using simple random sampling system, then three districts from each selected sub cities was nominated using a simple random sampling system. After the number of adolescent attending high school was identified in each selected districts, the study population was assigned proportionally to each district as per the number of adolescent students in high school in the district. Then, study participants were selected by using a systematic random sampling techniques.

Study variables

- Dependent Variable: Self-perception of physical appearance**.** ***Good self-perception:*** - refers to respondents who scored the mean and above the mean value of self-perception of one's physical appearance score. ***Poor self-perception:*** - refers to respondents who scored below the mean value of self-perception of one's physical appearance score.
- Independent Variables: Socio-demographic characteristics: Age, sex, class level, etc., Availability and accessibility of physical activities equipment in the school and nearby, Access to media (as source of information), Perception of self-dietary condition of the respondents

Data collection tools and quality control

Data were collected using structured questionnaire through interview based administration. The questionnaire was adapted from previous studies as it was appropriate for local context. The questionnaire was first prepared in English then translated to Amharic and then back to English to ensure consistency. The self-perception of one's physical appearance of the respondents was measured based on 17 items using likert scale measurement. Prior to the actual data collection, the questionnaire was pre-tested, then adjustment and correction was done based on the pretest result. To ensure data quality, adequate training and orientation were given to data collectors and supervisors. The completeness and appropriateness of the collected data were checked by supervisors every day and corrected according to the identified problems. In addition, the investigator monitored and evaluated the overall quality of the data collection process. Weight was measured to the nearest 0.1 kg, and height was measured to the nearest 0.1 cm. According to

WHO the BMI-for age >2SD: Obese; >1SD: Overweight; 1 - -2SD: Normal; <− 2 SD: Thinness; <− 3 SD: Severe thinness and height-for-age below -2SD is used for stunting (21).

Data analysis procedure

The data were entered into EPI data version 3.1 and then exported to SPSS version 23 for data management and analysis. Descriptive statistics of frequency, percentage, means and standard deviation were carried out. Logistic regression was employed to assess factors associated with self-perception of one's physical appearance. Accordingly, independent variables with $p<0.2$ during bivariate logistic regression were included to multivariable logistic regression analysis to determine the association between factors and good self-perception of one's physical appearance. Adjusted odds ratios with 95% confidence intervals and significance levels of $P< 0.05$ were used identify the determinant of good self-perception of one's physical appearance.

Ethics approval and consent to participate

Before conducting the study, ethical clearance was obtained from the Addis Ababa Health Bureau ethical clearance board and Addis Ababa educational bureau. Participants' right to self-determination and autonomy was respected, and study participants were given any information they needed verbally and in written prior to self-administration. For those who were below 18 years old, consent from their parents was obtained. The rights of each respondent refusing to answer a few or all questions were respected. Participation was voluntary, and participants could withdraw from the study at any time without explanation.

Results

Socio-demographic characteristics of respondents

A total of 283 adolescents participated in the study, with a response rate of 91.9%. About 144(50.9%) of respondents were male. The mean age of the respondents was 16.55 ± 1.2 (SD) years. The majority of respondents 116(41%) were attending grade 9. More than 92% of respondents had both parents (father and mother) alive. More than 70% monthly income level were less than 18000 ETB (Table 1).

Table 1. Distribution of socio-demographic characteristics of adolescents in Addis Ababa city, Ethiopia

Characteristics	Category	Frequency	Percent
Type of the school	Governmental	150	53
	Private	133	47
Sex of respondents	Male	144	50.9
	Female	139	49.1
Class level of the respondents	Grade 9	116	41
	Grade 10	82	82
	Grade 11	50	17
	Grade 12	35	12.4
Age of respondents in years	14-17 years	251	76
	18-19 Years	68	24
Is your parent alive?	Both father and mother is alive	263	92.9
	Only father is alive	6	2.1

	Only mother is alive	12	4.2
	Both father and mother is not alive	2	0.7
Do you live with your parent	Living with both parent	233	82.3
	Living with only one parent	37	13.1
	Living with relative	13	4.6
Father educational level	Illiterate	7	2.6
	Read and write	9	3.3
	Elementary	19	7.1
	High school	84	31.2
	Diploma	40	14.9
	>= first degree	110	40.9
Mother educational level	illiterate	10	3.6
	Read and write	16	5.8
	Elementary	22	8.0
	High school	93	33.5
	Diploma	63	22.9
	>= first degree	72	26.2
Mother occupation	Employer of (gov't & private org.)	74	26.9
	Merchant	65	23.6
	Daily laborer	21	7.6
	House wife	113	41.1
	Farmer	2	0.7
Father occupation	Employer of (gov't & private org.)	136	50.6
	Merchant	121	45.0

	Daily laborer	7	2.6
	Farmer	5	1.8
Family monthly income in ETB	<=18000	199	70.3
	>18000	84	29.7

Anthropometric measurement of respondents

Most of the respondents 177(62.5%) were within the normal range (18.5-24.9 kg/m^2) of body mass index, while approximately 19 (6.7%) were overweight (Table 2). The mean height of the respondents was 1.65 m with ± 0.095 (SD), while the mean weight of the respondents was 55.8 kg with ± 9.5 (SD).

Table 2: Anthropometric measurement of adolescents in Addis Ababa city, Ethiopia

**Variables**	**Category**	**Frequency**	**Percent**
BMI of respondents	<18.5 kg/m^2	87	30.7
	18.5-24.9 kg/m^2	177	62.5
	25-29.9 kg/m^2	19	6.7

Perception of dietary and related condition of the respondents

The overall good perception of dietary and related condition self-assessment of respondents were 55.5% and 44.6% had low perception. Based on the 5 level likert scale measurement of 19 items, the overall mean perception of respondents was 2.9 (±0.02). (Table 3).

Table 3: Mean perception of dietary and related condition of the adolescent in Addis Ababa city, Ethiopia

Characteristics	Mean (s.e.m)	Percent
Frightened of being overweight	4.13(0.076)	82.6
Aware of the calorie content of foods that I eat	1.88(0.067)	37.6
Particularly avoid food with high carbohydrate content	4.21(0.064)	0.842
Think burning up calories when i exercise	2.48(0.088)	49.6
Other people think that i am too thin	4.08(0.080)	81.6
Avoid food with much sugar	1.67(0.057)	33.4
Eat diet food	1.87(0.069)	37.4
I feel uncomfortable after eating sweets	4.3(0.068)	0.86
Engage in dieting behavior	1.61(0.054)	32.2
Feel that food control my life	1.89(0.074)	37.8
Give too much time and thought to food	1.89(0.073)	37.8
Have the urge to vomit after meals	4.65(0.044)	93
Avoid eating when I am hungry	1.49(0.052)	29.8
Cut my food into pieces	1.82(0.062)	36.4
Feel that others would prefer if I ate more	1.81(0.069)	36.2
I am preoccupied with the thought with of having fat on my body	1.72(0.065)	34.4
Take longer than other to eat my meal	4.3(0.059)	86
Display self-control around food	2.15(0.074)	43
Feel that others pressure me to eat	4.24(0.068)	84.8

Availability and accessibility of physical activity equipment

Among respondents, approximately 201 (71%) responded that there were regular physical activities in the school they were learning in. The majority 218(77%) of respondents responded that there was no gymnasium in the school. A total of 197 (69.6%) respondents had enough football field in the school they were learning in. About 203 (71.7%) respondents said there were sport clubs in the school. Approximately 155 (54.8%) and 145 (51.2%) respondents had regular physical activity in school and outside school, respectively (Table 4).

Table 4: Availability and accessibility of physical activity equipment for adolescents in, Addis Ababa city, Ethiopia

Characteristics	Possible options	Frequency	%
Availability of regular physical activity in the school they learn in	Yes	201	71
	No	82	29
Gymnasium access in the school	yes	65	23
	No	218	77
Availability of enough sport field in the school they learn in	Yes	197	69.6
	No	86	30.4
Availability enough sport field for sport in your district/near by	Yes	167	59
	No	116	41
Availability of sport club in your school	Yes	203	71.7
	No	80	28.3
Had regular physical activity in the school	Yes	155	54.8

	No	128	45.2
Had regular physical activity outside school	Yes	145	51.2
	No	138	48.8

Access to media as source of information

About 278 (98.5%) and 201 (71%) respondents had access to television and radio in their homes, respectively. Out of respondents, 241 (85.2%) had access to private mobile phones, while 193 (68.2%) had daily access to internet services. A total of 169 (59.7%) respondents accessed at least two types of social media daily. Approximately 151 (53.6%) and 201 (71%) respondents regularly followed Ethiopian and Western movies, respectively. Similarly, 130 (54.1%) and 157 (55.5%) respondents regularly followed Ethiopian and Western music's, respectively (Table 5).

Table 5: Media access of adolescents in Addis Ababa city, Ethiopia

**Characteristics**	**Possible options**	**Frequency**	**Percent**
Access to Television in their home	Yes	278	98.2
	No	5	1.8
Access to radio in their home	Yes	201	71
	No	82	29
Access to private mobile phone	Yes	241	85.2
	No	42	14.8
Access to internet daily	Yes	193	68.2
	No	90	31.8
Do use social media (FB, telegram, Este	Yes	169	59.7

gram, tweeter, you tube) at least two.	No	114	40.3
Follow Ethiopian movies/Cinemas regularly	Yes	151	53.6
	No	132	46.6
Follow western movies/Cinemas regularly	Yes	201	71
	No	82	29
Follow Ethiopian music's regularly	Yes	153	54.1
	No	130	45.9
Follow western music's regularly	Yes	157	55.5
	No	126	44.5

Level of self-perception of one's physical appearance

The overall good self-perception of one's physical appearance of respondents were 48.4% [95% CI=48.8, 541] (Fig 1).

Figure 1: The level of self-perception of one's physical appearance of adolescents in Addis Ababa city, Ethiopia.

Factors associated with self-perception physical appearance

Logistic regression was conducted to see the association of one independent variable with the dependent variables. Variables with p value <0.2 during the bivariate analysis were included in the multivariate logistic regression analysis to see the relative independent effect of each associated variable by controlling confounding variables. Accordingly, after managing for covariates, BMI of respondents, availability of enough football fields in the school they are learning in, daily access to internet service, following Ethiopian movies regularly, and following

Western music's regularly were significantly associated with adolescent self-perception of one's physical appearance (Table 6).

Table 6: Factors associated with self-perception of one's physical appearance of adolescents in, Addis Ababa city, Ethiopia

Variables		self-perception of physical appearance		COR [95% of CI]	AOR [95% of CI]
		Good Freq. (%)	Poor Freq. (%)		
Sex of respondents					
	Male	75(52.1)	69(47.9)	1.75(1.47, 2.21)*	1.00(0.59, 1.69)
	Female	82(59)	57(41)		1
Age of respondents					
	14-17 years	125(58.1)	90(41)	0.64(0.37, 1.11)	0.67(0.37, 1.22)
	18-19 Years	32(47.1)	36(52.9)	1	1
BMI of respondents					
	<18.5 kg/m^2	61(70.1)	26(29.9)	1	1
	18.5-24.9 kg/m^2	87(49.2)	90(50.8)	2.43(1.4, 4.9)**	2.56(1.45, 4.54)**
	25-29.9 kg/m^2	9(47.4)	10(52.6)	2.61(0.95, 7.16)	3.58(1.21, 10.56)*
Availability of enough sport field in the school you are learning in.					
	Yes	117(59.4)	80(40.6)	1.68(1.01, 2.80)*	1.89(1.09, 3.29)*
	No	40(46.5)	46(53.5)	1	1
Having regular physical activities outside their school					
	Yes	75(51.7)	70(48.3)	1.19(0.46, 1.17)	1.36(0.80, 2.29)

	No	82(59.4)	56(40.6)	1	1
Having Mobile phone					
	Yes	127(52.7)	114(47.3)	0.45(0.22, 0.91)*	1.48(0.62, 3.51)
	No	30(71.4)	12(28.6)	1	1
Daily access to internet service					
	Yes	98(50.8)	95(49.2)	1.54(1.32, 1.91)*	1.69(1.07, 2.94)*
	No	59(65.6)	31(34.4)	1	1
Following Ethiopian Movies/Cinemas regularly					
	Yes	96(63.6)	55(36.4)	2.03(1.26, 3.27)*	2.46(1.46, 4.15)**
	No	61(46.2)	71(53.8)	1	1
Following Western Movies/Cinemas regularly					
	Yes	106(52.7)	95(74.3)	1.68(1.2, 2.15)*	2.00(1.11, 3.59)*
	No	51(62.2)	31(37.8)	1	

*Significance at P<0.05, Significance at P<0.01

Discussion

This study aimed to assess the level of self-perception of physical appearance and its associated factors among adolescents in the Addis Ababa city, Ethiopia. Result of this study show that, the level of good self-perception of one body appearance of adolescents attending high school-level of education in Addis Ababa city was 48.6%. The finding of this study is lower than the finding of a similar study performed in Chile on the self-perception of physical fitness of Chilean adolescent students (3). Additionally, it is much lower than finding of a study done in Brazil on body image dissatisfaction among adolescents in public school students (9). However, the

finding is higher than that of a study performed in Romania (22). The difference might be due to differences in study settings in terms of development, culture and methodology used to assess self-perception.

Among the many determinants of self-perception of physical appearance, BMI plays an important role. Weight and height are the most important characteristics in adolescents that determine the self-perception of one's body (4). As such, the findings of this study also show that there is a strong association at $p<0.001$ between body mass index and self-perception of one physical appearance in adolescents. Compared to those with lower BMI, those in normal ranges were 2.56 times more likely to have a good perception of their physical appearance. This result is consistent with previous studies (2,9).

Having access to a suitable sport field is very important for children's physical development. Physical fitness is related to the ability to perform physical activity and evaluate one's body image (23). Adolescent physical self-esteem can be improved by participating in sports. Health benefits of exercise include increased aerobic power, increased muscle strength, and reduced obesity (24,25). As such, the results of this study all support this reality. Those who reported that there was enough football field and physical activity in the school had a highly good self-perception of their physical appearance. This result is consistent with a study performed in Canada (24). Similarly, it is supported by a review paper focused on school-based physical activity, body image, and academic performance.

The findings of this study show that there is a positive relationship between using internet services and self-perception of one's physical appearance. This means, compared to adolescents who did not have access to the internet, those who had internet access had a highly good self-

perception of their physical appearance. In contrast to this study, many findings show that there is a negative relationship between access to the internet and self-perception of one's physical appearance (26–28). Many researchers show that there is a strong relationship between internet access and self-perception of one's physical (28–31). Media pressure has been found to be the most powerful factor driving adolescent internalization. Information disseminated through mass media, especially online media, is often unrealistic, and body image research suggests that assimilation leads to discrepancies between actual and ideal body image, leading to lower self-esteem. has been shown (32).

Movies now make up the majority of media products people consume (33). It is the most common way of spending leisure time and has the greatest impact on modern life (34). Various studies have confirmed the many positive effects of movies on children and adolescents. One of them is the self-concept of appearance. This positive effect depends on the selection of films/movies appropriate for the developmental stages of children and adolescents (35,36). As such, the results of this study also show a strong relationship, $p < 0.01$, between self-perceptions of physical appearance and adherence/following of Ethiopian and Western films. However, some studies show that there is a negative relationship between adherence to cinema/films and self-perception of one's physical appearance (34,37).

Conclusion and recommendation

According to this study, only 48.4% of respondents reported a good self-perception of their physical appearance. This finding indicates that adolescents need to work to strengthen their good self-perception of their physical appearance. The results also show that, respondents' BMI, availability of enough sport fields in the school they are learning in, daily access to internet

service, following Ethiopian movies/cinemas regularly, following western music regularly are factors that significantly determine the good self-perception of one's physical appearance. Adolescents and their families need to work to achieve normal weight and height to develop a normal body mass index, which strengthens self-perceptions of their physical appearance. Schools need to increase access to enough sport fields to strengthen their physical fitness, which increases their self-perception of their physical appearance. Additionally, adolescents need to follow internet access, media, and movies/cinema selectively as they fit their developmental stage. Future research should consider well-organized studies that include many variables that enable the determination of underlying causes of poor self-perception of one's physical appearance.

Abbreviations

AOR: Adjusted Odds Ratio, BMI: Body Mass Index, CI: Confidence Interval, COR: Crude Odds Ratio, MUAC: Most Upper Arm Circumference, REC: Research and Ethical Committee, SD: Standard Deviation, SPSS: Statistical Package for Social Science

Declarations

Acknowledgement

My special thanks go to the Addis Ababa city health bureau all schools included in the study for their willingness to give me supportive letters and important information for my work. Last, I would like to thank all participants included in the study for their willingness to participate.

Funding

The authors received no specific funding for this work.

Availability of data and materials

The datasets used and/or analyzed during the current study is uploaded as Supporting Information file.

Consent for publication

Not Applicable.

Competing interest

The authors declare that they have no competing interests.

Reference

1. Riel CAHP Van, Bergh EEMM Den, Kemps HLM, Feuth T, Schreuder HWB, Hoogerbrugge PM, et al. Self-perception and quality of life in adolescents during treatment for a primary malignant bone tumour. Eur J Oncol Nurs [Internet]. 2014;18(3):267–72. Available at: http://dx.doi.org/10.1016/j.ejon.2014.01.005

2. Zadorozhnaya EG and L. Self-perception of Physical Appearance in Adolescents : Gender , Age and Self-perception of Physical Appearance in Adolescents : Gender , Age and Ethnic Aspects. Coll Antropol. 2016;40(2):73–81.

3. Vasquez P, Luarte-rocha C. Assessment of self-perception of physical fitness and proposal for standards among Chilean adolescent students : the EAPAF study. Arch Argent Pediatr. 2016;114(4):319–28.

4. Godina E, Zadorozhnaya L. Self-perception of Physical Appearance in Adolescents :

Gender , Age and Ethnic Aspects. Coll Antropol. 2016;40:73–81.

5. Tlaye KG, Belete MA, Demelew TM, Getu MA. Reproductive health services utilization and its associated factors among adolescents in Debre Berhan town , Central Ethiopia : a community-based cross-sectional study. 2018;1–11.

6. Tubić T, Đorđić V. Age and gender effects on global self-worth and domain-specific self-perceptions in youth. Tatjana Tubić and Višnja Đorđić. 2015;41–61.

7. Nicolosi S, Lipoma M. Self-esteem, physical self-perception and physical activities programs for adolescents. Stefan De Wals KM, redakteur. Rome: Nova Science Publishers, Inc; 2016. 299–310 bl.

8. Glauber Carvalho Nobre NCV. Self-perception of competence: concept, changes in childhood, and gender and age-group differences. J Phys Educ. 2019;30(2):1–10.

9. Santana LP, R RDC, Ana MO, Rosa M, Ester PC, Pinto DJ, et al. Factors associated with body image dissatisfaction among adolescents in public schools students in Salvador , Brazil. Nutr Hosp. 2013;28(3):747–55.

10. Al-zyoudi M. Gender differences in self-concept among adolescents with low vision. Int J Spec Educ. 2007;22(1):132–6.

11. Manning MA. Self-Concept and Self-Esteem in Adolescents [Internet]. Anne Arundel; 2007. Available at: www.naspcenter .org/principals

12. Esnaola I, Ses A. The Development of Multiple Self-Concept Dimensions During Adolescence. J Res Adolesc. 2018;30(2016):100–14.

13. Kelly LH. Florida State University Libraries The Relationship Between Physical Self-Concept , Body Image Dissatisfaction and Competition Anxiety in Female " Aesthetic " and " Non-Aesthetic " Collegiate Athletes. Florida State University; 2004.

14. Şahİn E, Barut Y, Ersanli E, Kumcağız H. Self-esteem and Social Appearance Anxiety : An Investigation of Secondary School Students. J Basic Appl Sci Res. 2014;4(3):152–9.

15. Moehlecke M, Blume CA, Cureau FV, Kieling C, Schaan BD. Self-perceived body image, dissatisfaction with body weight and nutritional status of Brazilian adolescents: a nationwide study. J Pediatr (Rio J). 2020;96(1):76–83.

16. da Silva AF, Moraes MS, Martins PC, Pereira EV, de Farias JM, Silva DAS. Prevalence of body image dissatisfaction and association with teasing behaviors and body weight control in adolescents. Motriz Rev Educ Fis. 2020;26(1):1–11.

17. Ribeiro-Silva R de C, Fiaccone RL, Conceição-Machado MEP da, Ruiz AS, Barreto ML, Santana MLP. Body image dissatisfaction and dietary patterns according to nutritional status in adolescents. J Pediatr (Rio J). 2018;94(2):155–61.

18. Weiss MR. Developmental Sport Psychology. Int Encycl Soc Behav Sci. 2001;3620–4.

19. Godina E. Self-perception of Physical Appearance in Adolescents : Gender , Age and Self-perception of Physical Appearance in Adolescents : Gender , Age and Ethnic Aspects. Coll Antropol. 2016;40(2):73–81.

20. Addis Ababa City adminstration Human resource management and development Bureau Annual report. 2019.

21. Cole TJ, Flegal KM, Nicholls D, Jackson AA. Body mass index cut offs to define thinness in children and adolescents: International survey. Br Med J. 2007;335(7612):194–7.

22. Pop CL. Association between body mass index and self body image perception. Iran J Public Health. 2017;46(12):1744–5.

23. Balsalobre FJB, Sánchez GFL, Suárez AD. Relationships between Physical Fitness and Physical Self-concept in Spanish Adolescents. Procedia - Soc Behav Sci [Internet]. 2014;132:343–50. Available at: http://dx.doi.org/10.1016/j.sbspro.2014.04.320

24. Bowker A. The relationship between sports participation and self-esteem during early adolescence. Can J Behav Sci. 2006;38(3):214–29.

25. Organized Sports for Children and Preadolescents. Pediatrics. 2001;107(6):1459–62.

26. Rohall DE, Cotten SR, Morgan C. Internet use and the self concept: Linking specific uses to global self-esteem. Curr Res Soc Psychol. 2002;8(1):1–18.

27. Hogan SR. The Effect of Social Media on Body Image Among Adolescents Results Methods. Calif State Univ. 2017;46(6).

28. Rodgers RF, Melioli T. The Relationship Between Body Image Concerns, Eating Disorders and Internet Use, Part I: A Review of Empirical Support. Adolesc Res Rev. 2016;1(2):95–119.

29. Hogue J V., Mills JS. The effects of active social media engagement with peers on body image in young women. Body Image [Internet]. 2019;28:1–5. Available at: https://doi.org/10.1016/j.bodyim.2018.11.002

30. Brown T, Champ J. Examining the Influence of Social Media on Body Image: Miss Perfection, a Misperception [Internet]. ProQuest Dissertations and Theses. Colorado State University; 2017. Available at: https://mountainscholar.org/handle/10217/183897

31. Lee HR, Lee HE, Choi J, Kim JH, Han HL. Social media use, body image, and psychological well-being: A cross-cultural comparison of korea and the united states. J Health Commun. 2014;19(12):1343–58.

32. You S. Body Esteem among Korean Adolescent Boys and Girls. Sustainability. 2019;11(2051):1–13.

33. Kubrak T. Impact of films: Changes in young People's attitudes after watching a movie. Behav Sci (Basel). 2020;10(5).

34. Mahmood I. Influence and Importance of Cinema on the Lifestyle of Educated Youth: A study on University Students of Bangladesh. IOSR J Humanit Soc Sci. 2013;17(6):77–80.

35. Javellana GM. Influence of Media on Body Image Satisfaction among Adolescents. Asia Pacific J Educ Arts Sci | [Internet]. 2014;1(1):95–101. Available at: http://apjeas.apjmr.com/wp-content/uploads/2014/04/APJEAS-2014-1-024.pdf

36. Agarwal V, Dhanasekaran S. Harmful effects of media on children and adolescents. J Indian Assoc Child Adolesc Ment Heal. 2012;8(2):38–45.

37. Hefner V, Woodward K, Figge L, Bevan JL, Santora N, Baloch S. The Influence of Television and Film Viewing on Midlife Women's Body Image, Disordered Eating, and Food Choice. Media Psychol. 2014;17(2):185–207.

Click here to access/download;Figure;Fig 1.tiff

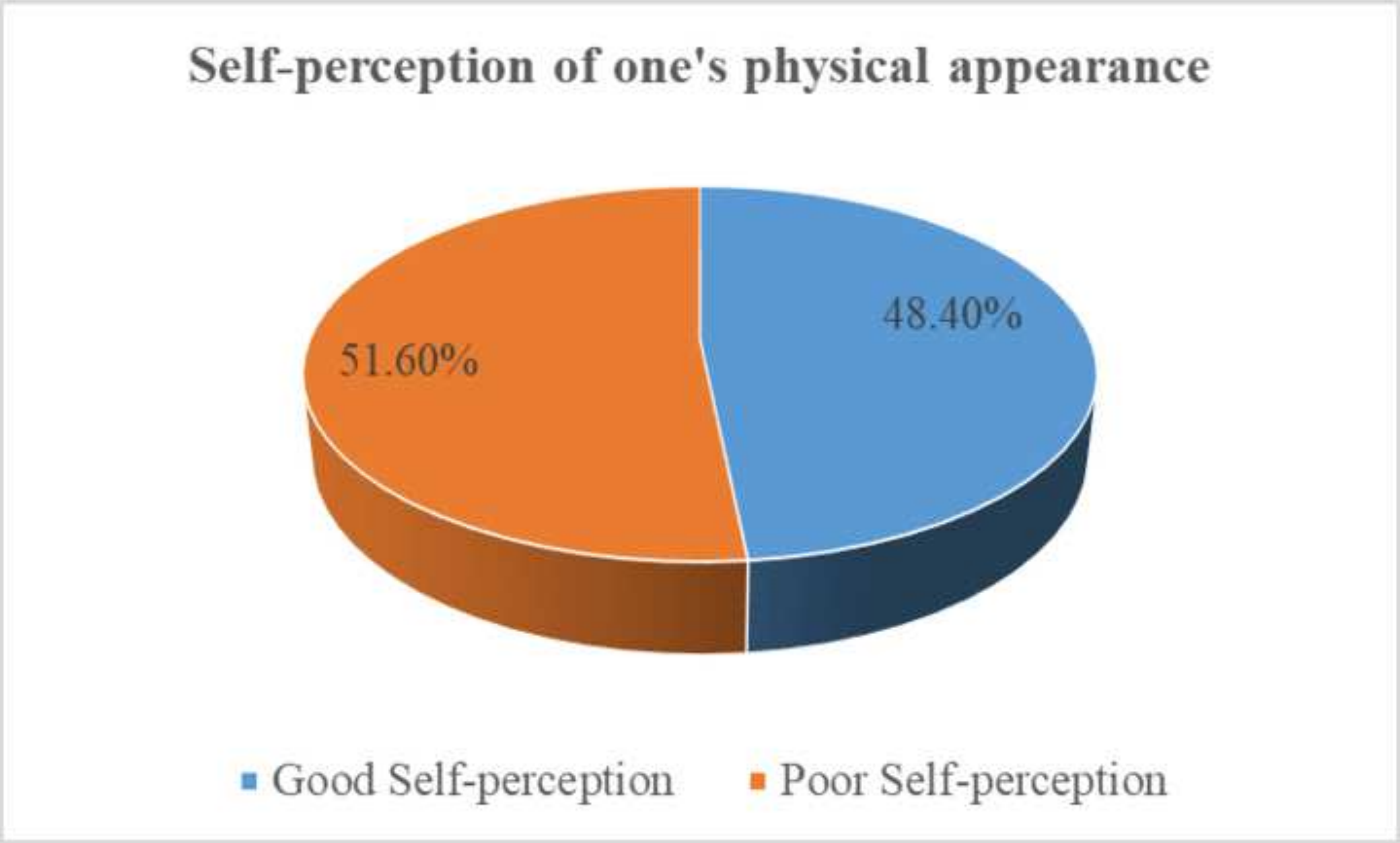

Click here to access/download
Supporting Information
Self-perception of physical apperance base line data.sav

Self-perception of ~~physical appearance~~Physical Appearance of ~~adolescents~~Adolescents and ~~associated factors~~Associated Factors in Addis Ababa, Ethiopia

Ziyad ~~Ahmed[1]~~Ahmed Abdo[1*], Semira Ahmed[2], Aynye Negese[3]

[1]Ethiopian Ministry of Health, Department of Hygiene and Environmental Health, Addis Ababa, Ethiopia. ~~Email: *ziyadahm1982@gmail.com*~~

[2]Ethiopian Ministry of Health, Department of Public Relation and Communication, Addis Ababa, Ethiopia. ~~Email: *ahmedsemira9@gmail.com*~~

[3]Ethiopian Ministry of Health, Department of Public Relation and Communication, Addis Ababa, Ethiopia. ~~Email: *aynyenegesse@gmail.com*~~

Corresponding author: Ziyad Ahmed Abdo, Email: *ziyadahm1982@gmail.com*

~~ABSTRACT~~

Abstract

Introduction~~:~~

Establishing a positive body image is a critical factor for adolescents' physical and mental health, as it ~~leads to~~determines self-confidence, sustainable individual growth and development throughout their lives. ~~There~~This reality needs to be supported by evidences generated locally. However, there is a ~~dearth~~lack of information in this regard in the study setting. ~~Therefore~~Thus,

Formatted: Font: 18 pt
Formatted: Line spacing: Double
Formatted: Font: 18 pt
Formatted: Font: 18 pt
Formatted: Font: 18 pt
Formatted: Font: 18 pt
Formatted: Font: Times New Roman
Formatted: Default Paragraph Font, Font: Calibri, 11 pt, Not Bold, Not Italic, Pattern: Clear
Formatted: Font: 16 pt, Font color: Auto
Formatted: Space After: 12 pt, Line spacing: Double

the aim of ~~this~~ study was to ~~assess the level of~~generate evidence by assessing self-perception of physical appearance and its associated factors among adolescents in Addis Ababa.

~~Method~~Methods and ~~material:~~ materials

A community-based cross-sectional study design was used to conduct the study. ~~A~~At the end of the multistage sampling procedure, a systematic random sampling technique was ~~implemented~~employed to select 308 study participants from selected districts. The questionnaire was adapted from previous studies as it was appropriate for local context. The data collectors were trained before the data collection and supervised during the data collection period, and the questionnaire was pretested. Logistic regression was employed to identify the predictor variables. Statistical significance was considered at P <0.05 with adjusted odds ratios calculated at 95% CIs.

Result~~:~~

The overall good self-perception of one's physical appearance was 48.4% [95% CI=48.8, 541]. Having BMI between 18.5 kg/m^2-24.9 kg/m^2 (AOR=2.56; 95% CI: 1.45, 4.54), presence of enough sport fields in the school (AOR=1.89; 95% CI: 1.09, 3.29), having daily access to internet services (AOR=1.69; 95% CI: 1.07, 2.94), following Ethiopian movies/cinemas (AOR=2.46; 95% CI: 1.46, 4.15), and regularly following ~~Western~~western movies/cinemas (AOR=2.0; 95% CI: 1.11, 3.59) were significantly associated with good self-perception of one's physical appearance.

Formatted: Font: 16 pt, Font color: Auto

Formatted: Line spacing: Double

Formatted: Heading 3 Char

Formatted: Font: 16 pt, Font color: Auto

Formatted: Space After: 12 pt, Line spacing: Double

Conclusion:

According to this study, good self-perception of one's physical appearance was low. Adolescents and their family need to work to bring normal body weight and height, which in turn brings their normal body mass index, which strengthensin turn will increase good self-perceptions of their physical appearance. Schools and the local administrations need to increase access to enough sport fields for students to strengthen their physical fitness, which increases their good self-perception of their physical appearance.

Key Words: Self-perception, Adolescent, Physical Appearance, Addis Ababa, Ethiopia

1. INTRODUCTION

Introduction

The problem of body image, its self-perception and dissatisfaction with one's own appearance is widely discussed in the anthropological and medical literatureliteratures (1–4).(1–4). Physical care and body image, especially induring adolescence, are social issues (5). Harter (1999) explainsdescribes self-perception as to evaluative judgments of attributes within discrete domains such as physical appearance, cognitive competence, athletic competence, social acceptance and so forth (6,7)(6,7). As suchThus, self-perception is developeddevelops through experience with thein social and physical environment, interactions with significant others and attributions of one's own behavior(8,9)., and individual behavioral traits (8,9). As children develop a sense of self andthemselves, interact with and gain experience in the world, and gain experiences, their self-

Formatted: Font: 16 pt, Font color: Auto

Formatted: Line spacing: Double

Formatted: Line spacing: Double

Formatted: Space After: 12 pt, Line spacing: Double

concept is affected. ~~Self-concept~~ is defined ~~as the value that an individual places on his or her own characteristics, qualities, abilities, and actions (10)~~(10).

The physical changes ~~experienced by~~ that the body ~~during the adolescent stage significantly influence the~~undergoes during adolescence have a profound impact on an individual's personal and social identity ~~of the individual~~ (11). For this reason, the process of forming physical self-perception can be considered inherent to this age and can also influence physical ~~sports~~exercise habits (5,12). From the above, it is possible to conclude that a good body image can be associated with ~~a good~~better self-perception ~~and, likewise, with~~, as well as a more active and less sedentary lifestyle~~. Adolescents experience physical and psychosocial changes as part of their normal development~~ ~~(1).~~(1). These interpretations are also influenced by significant others' evaluations, reinforcements, and attributions of the individual's behavior (11,13).

Formatted: Space After: 12 pt, Line spacing: Double

~~School is an important level of education in which adolescents continue to learn the minimum knowledge and skills that every citizen must possess (14). It is a transition to a new educational level and can be stressful for adolescents since they encounter a new curriculum, possibly new and different friends, new teachers, preparation for an important and life-changing exam, and entering puberty (15). Specifically, at the secondary educational level, adolescents gain problem solving skills, develop accordance with social values, and basic abilities for functioning in society (14). In addition, this educational process plays a crucial role in the development of self-perception of their physical appearance in adolescents.~~

~~Studies indicate that the prevalence of body image dissatisfaction in developed countries ranges between 35% and 81% among female adolescents and between 16% and 55% among male adolescents(9,16–18). Despite the evidence-based on data from developed countries, knowledge about the prevalence of body image dissatisfaction and associated factors during adolescence in developing countries such as Ethiopia is scarce (23). Some evidence indicates that biological~~

~~factors, including age, sex, puberty, and body composition, along with psychological factors, including depression, low self-esteem, and adoption of weight-loss strategies, are conditions of risk for body image dissatisfaction during adolescence (17).~~

~~During adolescence, people~~School is an important level of education in which adolescents continue to learn the minimum level of knowledge and skills that every citizen should have (9,14). Entering to high school is a transition to a new educational level and can be stressful for adolescents since they encounter a new curriculum, possibly new and different friends, new teachers, preparation for an important and life-changing exam, and entering puberty (14). Specifically, at the secondary level of education, adolescents acquire problem-solving skills and develop them according to social values and basic skills for functioning in society(3). In addition, this educational process plays a crucial role in the development of self-perception of their physical appearance in adolescents.

Studies have shown that the prevalence of body image dissatisfaction in developed countries ranges from 35% to 81% in adolescent females and 16% to 55% in adolescent males (9,15–17). Despite data-based evidence from developed countries, little is known about the prevalence of adolescent body image and related factors of dissatisfaction in developing countries such as Ethiopia (23).

Several lines of evidence suggest that biological factors such as age, sex, puberty, body composition, and psychological factors such as depression, low self-esteem, and adoption of weight loss strategies are factors for adolescent body image dissatisfaction (17). parents also affect adolescent self-perception through emotion, motivation, beliefs, values, expectancies, and behaviors (18). A study also show that, self-perception of one’s body, weight was the most

important characteristics in adolescent (19). Peers are central to children's psychological and self-image development and (18).

Adolescents face ~~different challenging issues~~many challenges, such as ~~establishing~~solidifying their personality, accepting physical changes, ~~separation from family~~leaving their families, building norms and moral~~ rules and~~ values, choosing a ~~profession~~career, and becoming a contributing ~~members~~member of society (17). These serious developmental ~~tasks~~challenges and ~~possible stress~~potential sources of stress can be ~~harmful~~detrimental to the development of healthy self-perception and positive body image (24). ~~For healthy generations, experiencing healthy adolescence is very important.~~ High self-perception and a positive body image ~~positively affect an individual's~~have a positive impact on an individual's quality of life, ~~achievements~~performance, and~~ personal~~ relationships. Adolescents who fail to develop a positive body image and self-perception are at risk of depression~~ (25).~~, other health condition and related personal problems (25).

Formatted: Space After: 12 pt, Line spacing: Double

Although the level of satisfaction with the self-perception of body image of adolescents is a reason for the growing attention of national and international organizations related to education and health promotion, governments, and researchers (19), there are a limited number of studies related to the self-perception of the physical appearance of adolescents, and little is known about the factors related to ~~dissatisfaction~~satisfaction with one's body image. Similarly, to our knowledge, there is no study done on this issue in Ethiopia, specifically in Addis Ababa. As such, this study intends to ~~assess~~provide evidence by assessing the magnitude of self-perception of physical appearance and its associated factors among adolescents in Addis Ababa, Ethiopia.

Formatted: Space After: 12 pt, Line spacing: Double

~~2. METHDOLOGY~~

Materials and Methods

~~2.1.~~ Study setting and design

A community-based cross-sectional study design was used to conduct the study. The study was conducted in Addis Ababa, Ethiopia. Addis Ababa is the capital city and largest city of Ethiopia and also the country's political, economic, cultural and tourism center. It is located in the central part of Ethiopia and close to the equator. Sitting at the foot of mount Entoto at an elevation of 2,355 meter~~, Addis Ababa is the highest city in Africa. It cover an area of 527 km^2 and has a population size of about 4 million(19).~~. It cover an area of 527 km^2 and has a population size of about 4 million (20).

~~2.2.~~ Population and eligibility criteria

The source population of this study was all adolescents in Addis Ababa city who ~~attended~~was attending high school education. The study population was all adolescents in selected districts and attending high school in ~~Kolfe Keranio and Yeka~~selected sub ~~city~~cities. All selected adolescent students in selected districts who were in high school education during the data collection time and in the adolescent age group (10-19 years) were included in the study. Adolescents with chronic health problems, especially those with diabetes, were excluded from the study.

Formatted: Font: 16 pt, Font color: Auto

Formatted: Font: 16 pt, Not Bold

Formatted: Heading 2, Space After: 12 pt, No bullets or numbering

Formatted: Space After: 12 pt, Line spacing: Double

Formatted: Heading 2, Space After: 12 pt, Line spacing: single, No bullets or numbering

Formatted: Font: 16 pt, Font color: Auto

Formatted: Font: 16 pt, Not Bold

Formatted: Space After: 12 pt, Line spacing: Double

~~2.3.~~ Sample size ~~and sampling strategy~~determination

The required sample size for ~~the~~this study was calculated by using a single population proportion formula with the following assumptions: 95% confidence level, 5% margin of error and 19.5% proportion of satisfaction with one's physical appearance taken from a previous study performed in Brazil (9).

$$n = \frac{(1.96)^2\ 0.195(1-0.195)}{(0.05)^2} = 280$$

By considering 10% non-response rate, a total of 308 sample populations was included.

Sampling techniques

Multistage sampling techniques were used to select study participants. First Kolfe Keranio and Yeka sub city was selected from ~~subsidy~~sub-cities in Addis Ababa using simple random sampling system, then three districts from each selected sub ~~city~~cities was ~~selected~~nominated using a simple random sampling system. ~~The~~After the number of adolescent attending high school was identified in each selected districts, the study population was assigned proportionally to each ~~selected~~ district as per the number of adolescent students in high school in ~~each~~the district. Then, study participants were selected by using a ~~simple~~systematic random sampling ~~system.~~techniques.

~~2.4.~~ Study variables

- ~~**Dependent Variable:** Level of self-perception of physical appearance~~
- Dependent Variable: Self-perception of physical appearance**. *Good self-perception:*** - refers to respondents who scored the mean and above the mean value of self-perception of one's

Formatted: Font: 16 pt, Font color: Auto

Formatted: Heading 2, Space After: 12 pt, No bullets or numbering, Adjust space between Latin and Asian text, Adjust space between Asian text and numbers

Formatted: Font: 16 pt, Not Bold, Font color: Auto

Formatted: Line spacing: Double

Formatted: Line spacing: Double

Formatted: Font: Times New Roman, 16 pt, Font color:

Formatted: Font: Times New Roman, 16 pt

Formatted: Heading 2, Space After: 12 pt, No bullets or numbering

physical appearance score. ***Poor self-perception:*** - refers to respondents who scored below the mean value of self-perception of one's physical appearance score.

- Independent Variables: Socio-demographic characteristics: Age, sex, class level, etc., Availability and accessibility of physical activities equipment in the school and nearby, Access to media (as source of information), Perception of self-dietary condition ~~and related~~ of the respondents

~~2.5.~~ Data collection tools and quality control

Data were collected using ~~an interviewer-administered~~ structured questionnaire ~~that was~~ through interview based administration. The questionnaire was adapted from previous studies~~. Prior to the actual data collection, the questionnaire was adjusted and corrected based on the pretest result, and the final questionnaire was~~ as it was appropriate for local context. The questionnaire was first prepared in English then translated to Amharic and then back to English to ensure consistency. ~~Data were collected by 4 health extension workers and supervised by 2 experienced public health specialists.~~The self-perception of one's physical appearance of the respondents was measured based on 17 items using likert scale measurement. Prior to the actual data collection, the questionnaire was pre-tested, then adjustment and correction was done based on the pretest result. To ensure data quality, adequate training and orientation were given to data collectors and supervisors. The completeness and appropriateness of the collected data were checked by supervisors every day and corrected according to the identified problems. In addition, the investigator monitored and evaluated the overall quality of the data collection process. ~~The questionnaire was pretested and modified based on the findings related to clarity, wordings, logical sequence, skip patterns of the questions and resources needed.~~ Weight was measured to the nearest 0.1 kg, and height was measured to the nearest 0.1 cm. ~~Mean~~ According to WHO the BMI-for age >2SD: Obese; >1SD: Overweight; 1 - -2SD: Normal; <− 2 SD: Thinness; <− 3 SD: Severe thinness and height ~~and weight were computed, and BMI (weight (kg)/height (m2) was calculated. MUAC was also measured by standard measurement.~~

Formatted: List Paragraph,Bullets,Heading II,List Paragraph (numbered (a)),List Paragraph1,List Paragraph11,List bullet,Numbered List Paragraph,References, Line spacing: Double, Bulleted + Level: 1 + Aligned at: 0" + Indent at: 0.25", Don't adjust space between Latin and Asian text, Don't adjust space between Asian text and numbers

Formatted: Font: Bold, Font color: Auto

Formatted: Font: 16 pt, Font color: Auto

Formatted: Font: 16 pt, Not Bold, Font color: Auto

Formatted: Heading 2, Space After: 12 pt, No bullets or numbering

Formatted: Font color: Black

Formatted: Font color: Auto

2.6. Operational definition

Good self-perception: - refers to respondents who scored the mean and above the mean value of self-perception one's physical appearance score. **Low self-perception:** - refers to respondents who scored-for-age below the mean value of self-perception one's physical appearance score.-2SD is used for stunting (21).

2.7. Data analysis procedure

The data were entered into EPI data version 3.1, which is epidemiological software packages for editing, cleaning, coding, and check completeness and consistency, and then exported to SPSS version 23 for data management and analysis. Descriptive statistics of frequency, percentage, means and standard deviation were carried out. In addition, bivariate analysis Logistic regression was used to identify significant variables, andemployed to assess factors associated with self-perception of one's physical appearance. Accordingly, independent variables with p<2.0 were fitted to binary 0.2 during bivariate logistic regression were included to multivariable logistic regression analysis to determine the association between factors and good self-perception of one's physical appearance. OddsAdjusted odds ratios with 95% confidence intervals and significance levels atof P< 0.05 were used to assessidentify the association between factors and the leveldeterminant of good self-perception of one's physical appearance. An adjusted odds ratio with a 95% confidence interval was calculated. The output of the analysis is displayed in statistical tables.

Formatted: Line spacing: Double

Formatted: Font: 16 pt, Font color: Auto

Formatted: Font: 16 pt, Not Bold

Formatted: Heading 2, Space After: 12 pt, No bullets or numbering, Adjust space between Latin and Asian text, Adjust space between Asian text and numbers

Formatted: Line spacing: Double

Formatted: English (United Kingdom)

~~2.8.~~ Ethics approval and consent to participate

Before conducting the study, ethical clearance was obtained from the Addis Ababa Health Bureau ethical clearance board and Addis Ababa educational bureau. Participants' right to self-determination and autonomy was respected, and study participants were given any information they needed verbally and in written prior to self-administration. For those who were below 18 years old, consent from their parents was obtained. The rights of each respondent refusing to answer a few or all questions were respected. Participation was voluntary, and participants could withdraw from the study at any time without explanation.

~~3. RESULTS~~

Results

~~3.1.~~ Socio-demographic characteristics of respondents

A total of 283 adolescents participated in the study, with a response rate of 91.9%. ~~Among respondents, 150 (53%) were those who attended governmental school. Approximately~~About 144(50.9%) of respondents were male. The mean age of the respondents was 16.55 ± 1.2 (SD) years. The majority of respondents 116(41%) were attending grade 9~~, followed by those attending grade 10. More than 92% of respondents had both parents (father and mother) alive. From this, the majority 233 (82.3%) of respondents were living with both of their parents. Approximately 110 (40.9%) of the respondents' father's educational level was a first degree or above. Most 93 (33.5%) of the respondents' mothers had a high school educational level. However, most 113 (41.8%) of respondents' mother occupations were house wives. Similarly, approximately 136 (50.6%) of~~

Formatted: Font: 16 pt, Font color: Auto

Formatted: Font: 16 pt, Not Bold

Formatted: Heading 2, Space After: 12 pt, No bullets or numbering

Formatted: Line spacing: Double

Formatted: Font: 16 pt, Font color: Auto

Formatted: Heading 2, Space After: 12 pt, Line spacing: single, No bullets or numbering

Formatted: Font: 16 pt, Not Bold

Formatted: Line spacing: Double

~~respondents' father occupations were employers at either governmental or private organizations, followed by merchants. See Table 1.~~. More than 92% of respondents had both parents (father and mother) alive. More than 70% monthly income level were less than 18000 ETB (Table 1).

Table 1~~:~~. Distribution of socio-demographic characteristics of adolescents in Addis Ababa city, Ethiopia

**Characteristics**	**Category**	**~~frequency~~Frequency**	**~~%~~Percent**
Type of the school	Governmental	150	53
	Private	133	47
Sex of respondents	Male	144	50.9
	Female	139	49.1
Class level of the respondents	Grade 9	116	41
	Grade 10	82	82
	Grade 11	50	17
	Grade 12	35	12.4
Age of respondents in years	14-17 years	251	76
	18-19 Years	68	24
Is your parent alive?	Both father and mother is alive	263	92.9
	Only father is alive	6	2.1
	Only mother is alive	12	4.2
	Both father and mother is not alive	2	0.7
	Living with both parent	233	82.3
	Living with only one parent	37	13.1

Do you live with your parent	Living with relative	~~11~~13	4.~~7~~6
Father educational level	Illiterate	7	2.6
	Read and write	9	3.3
	Elementary	19	7.1
	High school	84	31.2
	Diploma	40	14.9
	>= first degree	110	40.9
Mother educational level	illiterate	10	3.6
	Read and write	16	5.8
	Elementary	22	8.0
	High school	93	33.5
	Diploma	63	22.9
	>= first degree	72	26.2
Mother occupation	Employer of (gov't & private org.)	74	26.9
	Merchant	65	23.6
	Daily laborer	21	7.6
	House wife	113	41.1
	Farmer	2	0.7
Father occupation	Employer of (gov't & private org.)	136	50.6
	Merchant	121	45.0
	Daily laborer	7	2.6
	Farmer	5	1.8
	<=18000	199	70.3

Formatted: Line spacing: Double

Formatted: Line spacing: Double

Formatted: Line spacing: Double

Formatted: Line spacing: Double

Formatted: Line spacing: Double

Family monthly income in ETB	>18000	84	29.7

3.2. Anthropometric measurement of respondents

Most of the respondents 177(62.5%) were within the normal range (18.5-24.9 kg/m^2) of body mass index, while approximately 19 (6.7%) were overweight. Concerning middle upper arm circumstances, 55 (38.5%) male respondents' MUACs were within the normal range (23-24.9 cm) for adolescents, followed by the overweight range. Approximately 75 (54%) female respondents were within the normal range (22-23.9 cm) of MUAC levels. (Table 2). The mean height of the respondents was 1.65 m with ± 0.095 (SD), while the mean weight of the respondents was 55.8 kg with ± 9.5 (SD). See Table 2.

Table 2: Anthropometric measurement of adolescents in Addis Ababa city, Ethiopia

Variables	Category	Frequency	%Percent
BMI of respondents	<18.5 kg/m^2	87	30.7
	18.5-24.9 kg/m^2	177	62.5
	25-29.9 kg/m^2	19	6.7
Mid-Upper arm circumferences			
Male	<23 cm	59	41
	23-24.9 cm	55	38.2
	>25 cm	30	20.8
Female	<22 cm	31	22.3
	22-23.9 cm	75	54
	>24Cm	33	23.7

Formatted: Line spacing: Double
Formatted: Font: 1 pt, English (United States)
Formatted: Heading 2, Space After: 12 pt, Line spacing: Double
Formatted: Font: 16 pt
Formatted: Line spacing: Double, No bullets or numbering
Formatted: Line spacing: Double
Formatted: Font color: Auto
Formatted Table
Formatted: Font: 12 pt
Formatted: Line spacing: Double

3.3. Perception of dietary and related condition of the respondents

According to responses rated based on five Likert scales, namely, 1-strongly disagree, 2-disagree, 3-not sure, 4-agreeThe overall good perception of dietary and 5-strongly agreerelated condition self-assessment of respondents were 55.5% and 44.6% had low perception. Based on the 5 level likert scale measurement of 19 items, the overall mean perception of respondents was 2.9 (±0.02). Based on the definition of good and low perception, approximately 157 (55.5%) respondents had good perception, and 126 (44.6%) had low(Table 3).

Table 3: Mean perception, of dietary and related condition of the adolescent in Addis Ababa city, Ethiopia

Characteristics	Mean (s.e.m)	Percent
Frightened of being overweight	4.13(0.076)	82.6
Aware of the calorie content of foods that I eat	1.88(0.067)	37.6
Particularly avoid food with high carbohydrate content	4.21(0.064)	0.842
Think burning up calories when i exercise	2.48(0.088)	49.6
Other people think that i am too thin	4.08(0.080)	81.6
Avoid food with much sugar	1.67(0.057)	33.4
Eat diet food	1.87(0.069)	37.4
I feel uncomfortable after eating sweets	4.3(0.068)	0.86
Engage in dieting behavior	1.61(0.054)	32.2
Feel that food control my life	1.89(0.074)	37.8

Formatted: Font: 1 pt
Formatted: Heading 2, Line spacing: Double
Formatted: Font: 16 pt
Formatted: Line spacing: Double, No bullets or numbering
Formatted: Font: 12 pt
Formatted: Caption, Keep with next, Adjust space between Latin and Asian text, Adjust space between Asian text and numbers
Formatted: Font: 10 pt

Give too much time and thought to food	1.89(0.073)	37.8
Have the urge to vomit after meals	4.65(0.044)	93
Avoid eating when I am hungry	1.49(0.052)	29.8
Cut my food into pieces	1.82(0.062)	36.4
Feel that others would prefer if I ate more	1.81(0.069)	36.2
I am preoccupied with the thought with of having fat on my body	1.72(0.065)	34.4
Take longer than other to eat my meal	4.3(0.059)	86
Display self-control around food	2.15(0.074)	43
Feel that others pressure me to eat	4.24(0.068)	84.8

~~3.4.~~ Availability and accessibility of physical activity equipment ~~in the school and nearby~~

Among respondents, approximately 201 (71%) responded that there were regular physical activities in the school they were learning in. The majority ~~(~~218~~,~~ (77%) of respondents responded that there was no gymnasium in the school. A total of 197 (69.6%) respondents had enough football field in the school they were learning in. ~~Approximately~~About 203 (71.7%) respondents said there were sport clubs in the school. Approximately 155 (54.8%) and 145 (51.2%) respondents had regular physical activity in school and outside school, respectively~~. See~~ (Table ~~3.~~4).

Table ~~3~~4: Availability and accessibility of physical activity equipment for adolescents in, Addis Ababa city, Ethiopia

Characteristics	Possible options	Frequency	%
	Yes	201	71

Formatted: Font: 16 pt

Formatted: Line spacing: Double, No bullets or numbering

Formatted: Font: 16 pt

Formatted: Space After: 12 pt, Line spacing: Double

Formatted: Line spacing: Double

Formatted Table

Availability of regular physical activity in the school they learn in	No	82	29
Gymnasium access in the school	yes	65	23
	No	218	77
Availability of enough sport field in the school they learn in	Yes	197	69.6
	No	86	30.4
Availability enough sport field for sport in your district/near by	Yes	167	59
	No	116	41
Availability of sport club in your school	Yes	203	71.7
	No	80	28.3
Had regular physical activity in the school	Yes	155	54.8
	No	128	45.2
Had regular physical activity outside school	Yes	145	51.2
	No	138	48.8

~~3.5.~~ Access to media as source of information

~~A majority of~~About 278 (98.5%) and 201 (71%) respondents had access to television and radio in their homes, respectively. Out of respondents, 241 (85.2%) had access to private mobile phones, while 193 (68.2%) had daily access to internet services. A total of 169 (59.7%) respondents accessed at least two types of social media daily. Approximately 151 (53.6%) and 201 (71%) respondents regularly followed Ethiopian and Western movies, respectively. Similarly, 130

Formatted: Line spacing: Double

Formatted: Line spacing: Double

Formatted: Font: 12 pt

Formatted: Line spacing: Double

Formatted: Line spacing: Double

Formatted: Line spacing: Double

Formatted: Line spacing: Double

Formatted: Line spacing: Double

Formatted: Line spacing: Double

Formatted: Font: 16 pt

Formatted: Space Before: 0 pt, Line spacing: Double, No bullets or numbering

Formatted: Line spacing: Double

(54.1%) and 157 (55.5%) respondents regularly followed Ethiopian and Western ~~movies~~music's, respectively~~. See ~~ (Table ~~4.~~5).

Table ~~4~~5: Media access of adolescents in Addis Ababa city, Ethiopia

Characteristics	Possible ~~Options~~ options	Frequency		Percent ~~%~~
Access to Television in their home	~~yes~~Yes	278		98.2
	No	5		1.8
Access to radio in their home	Yes	201		71
	No	82		29
Access to private mobile phone	~~yes~~Yes	241		85.2
	No	42		14.8
Access to internet daily	~~yes~~Yes	193		68.2
	No	90		31.8
Do use social media (FB, telegram, Este gram, tweeter, you tube) at least two.	~~yes~~Yes	169		59.7
	No	114		40.3
Follow Ethiopian movies/Cinemas regularly	Yes	151		53.6
	No	132		46.6
Follow western movies/Cinemas regularly	~~yes~~Yes	201		71
	No	82		29
	~~yes~~Yes	153		54.1

Follow Ethiopian music's regularly	No	130		45.9
Follow western music's regularly	yesYes	157		55.5
	No	126		44.5

3.6. Level of self-perception of one's physical appearance

The overall mean level of self-perception of the physical appearance of respondents was 2.18 (±0.35). Based on the definition of good and low levels of self-perception, approximately 48.4% [95% CI=48.8, 541] of respondents had good self-perception, and 51.6% [95% CI=45.9, 57.2] had low self-perception of one's physical appearance. See Figure of respondents were 48.4% [95% CI=48.8, 541] (Fig 1.).

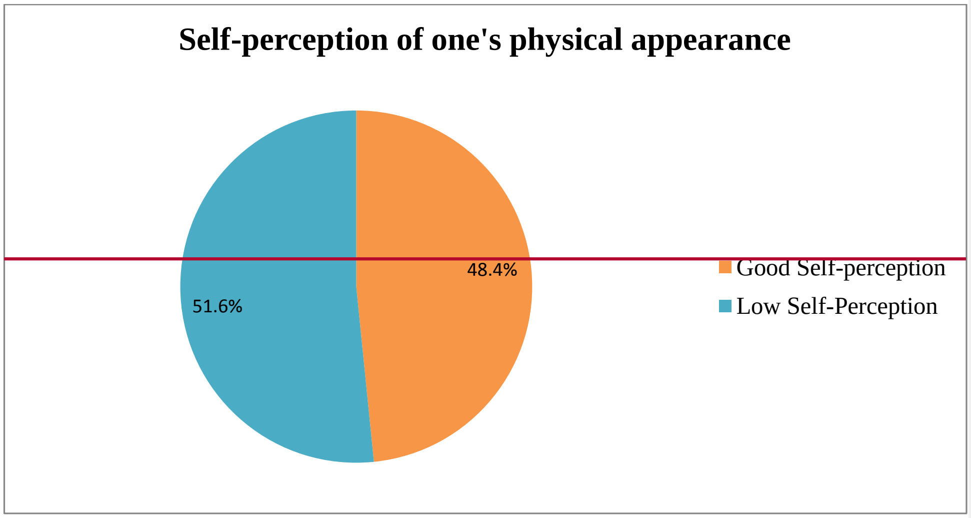

Figure 1: MeanThe level of self-perception of one's physical appearance of adolescents in Addis Ababa city, Ethiopia.

Formatted: Line spacing: Double
Formatted: Line spacing: Double
Formatted: Line spacing: Double
Formatted: Line spacing: Double
Formatted: Line spacing: Double
Formatted: Line spacing: Double
Formatted: Font: 16 pt
Formatted: Line spacing: Double, No bullets or numbering
Formatted: Line spacing: Double
Formatted: Line spacing: Double

~~3.7.~~ Factors associated with self-perception physical appearance

Logistic regression was conducted to see the association of one independent variable with the dependent variables. Variables with ~~a~~ p value <0.2 during the bivariate analysis were included in the multivariate logistic regression analysis to see the relative independent effect of each associated variable by controlling confounding variables. Accordingly, after managing for covariates, BMI of respondents, availability of enough football fields in the school they are learning in, daily access to internet service, following Ethiopian movies regularly, and following Western ~~movies~~music's regularly were significantly associated with adolescent self-perception of one's physical appearance~~. See~~ (Table ~~5.~~6).

Table ~~5~~6: Factors associated with self-perception of one's physical appearance of adolescents in, Addis Ababa city, Ethiopia

**Variables**	**~~Level of~~ self-perception of physical appearance**		**COR [95% of CI]**	**AOR [95% of CI]**
	**Good Freq. (%)**	**~~Low~~ Poor Freq. (%)**		
~~Type of the school attending~~				
~~Government~~	~~84(56.7)~~	~~65(43.3)~~	~~1.11(0.69, 1.77)~~	~~0.84(0.50, 1.43)~~
~~Private~~	~~72(54.1)~~	~~61(45.9)~~	~~1~~	~~1~~
Sex of respondents				
Male	75(52.1)	69(47.9)	1.75(1.47, 2.21)*	1.00(0.59, 1.69)
Female	82(59)	57(41)		1
Age of respondents				

Formatted: Font: 16 pt
Formatted: Line spacing: Double, No bullets or numbering
Formatted: Line spacing: Double
Formatted: Font: Bold
Formatted: Line spacing: Double
Formatted: Font: Bold
Formatted: Font: Bold
Formatted Table
Formatted: Font: 12 pt
Formatted: Line spacing: Double
Formatted: Font: 12 pt
Formatted: Line spacing: Double
Formatted Table
Formatted: Font: 12 pt
Formatted: Line spacing: Double
Formatted: Font: 12 pt
Formatted: Line spacing: Double
Formatted: Font: 12 pt
Formatted: Line spacing: Double

14-17 years	125(58.1)	90(41)	0.64(0.37, 1.11)	0.67(0.37, 1.22)
18-19 Years	32(47.1)	36(52.9)	1	1
BMI of respondents				
<18.5 kg/m^2	61(70.1)	26(29.9)	1	1
18.5-24.9 kg/m^2	87(49.2)	90(50.8)	2.43(1.4, 4.9)**	2.56(1.45, 4.54)**
25-29.9 kg/m^2	9(47.4)	10(52.6)	2.61(0.95, 7.16)	3.58(1.21, 10.56)*
Availability of enough sport field in the school you are learning in.				
Yes	117(59.4)	80(40.6)	1.68(1.01, 2.80)*	1.89(1.09, 3.29)*
No	40(46.5)	46(53.5)	1	1
Having regular physical activities outside their school				
Yes	75(51.7)	70(48.3)	1.19(0.46, 1.17)	1.36(0.80, 2.29)
No	82(59.4)	56(40.6)	1	1
Having Mobile phone				
Yes	127(52.7)	114(47.3)	0.45(0.22, 0.91)*	1.48(0.62, 3.51)
No	30(71.4)	12(28.6)	1	1
Daily access to internet service				
Yes	98(50.8)	95(49.2)	1.54(1.32, 1.91)*	1.69(1.07, 2.94)*
No	59(65.6)	31(34.4)	1	1
Following Ethiopian Movies/Cinemas regularly				
Yes	96(63.6)	55(36.4)	2.03(1.26, 3.27)*	2.46(1.46, 4.15)**
No	61(46.2)	71(53.8)	1	1
Following Western Movies/Cinemas regularly				
Yes	106(52.7)	95(74.3)	1.68(1.2, 2.15)*	2.00(1.11, 3.59)*

	No	51(62.2)	31(37.8)	1	

*Significance at P<0.05, Significance at P<0.01,

4. DISCUSSION

Discussion

This study aimed to assess the level of self-perception of physical appearance and its associated factors among adolescents in the Addis Ababa city, Ethiopia. Result of this study show that, the level of good self-perception of one body appearance of adolescents attending high school-level education in Addis Ababa city was 48.6%. The findings of this study are lower than the findings of a similar study performed in Chile on the self-perception of physical fitness of Chilean adolescentof education in Addis Ababa city was 48.6%. The finding of this study is lower than the finding of a similar study performed in Chile on the self-perception of physical fitness of Chilean adolescent students (3). Additionally, it is much lower than finding of a study done in Brazil on body image dissatisfaction among adolescents in public school students (9). Additionally, it is much lower than a study done in Brazil on body image dissatisfaction among adolescents in public school students(9). However, the finding is higher than that of a study performed in Romania(20).However, the finding is higher than that of a study performed in Romania (22). The difference might be due to differences in study settings in terms of development, culture and methodology used to assess self-perception.

Among the many determining factors for self-perceptiondeterminants of one's self-perception of physical appearance, BMI plays a majoran important role. In self perception of one's body, weightWeight and height are the most important characteristics in adolescents that determine the

Formatted: Font: 12 pt

Formatted: Line spacing: Double

Formatted: Space After: 12 pt, Line spacing: Double

Formatted: Line spacing: Double

Formatted: Space After: 12 pt, Line spacing: Double

self-perception of one's body (4). As such, the findings of this study also show that there is a strong association (at $p<0.001$) between body mass index and self-perception of one physical appearance in adolescents. Compared to those with lower BMI, those in normal ranges were 2.56 times more likely to have a good perception of their physical appearance. This result is consistent with previous studies (2,9).

~~The availability of enough football fields is very important for the development of physical fitness in boys. Physical fitness is related to the ability to perform physical activity and evaluate one's body image (21). Physical self-esteem may be enhanced during adolescence through sports involvement. The health benefits of sports include increased aerobic power, increased muscle strength, and decreased obesity(22,23). As such, the results of this study all support this reality. Those who reported that there was enough football field and physical activity in the school had a highly good self-perception of their physical appearance. This result is consistent with a study performed in Canada (22). Similarly, it is supported by a review paper focused on school-based physical activity, body image, and academic performance.~~

~~The findings of this study show that there is a positive relationship between using internet services and self-perception of one's physical appearance. This means that compared to adolescents who did not have access to the internet, those who had internet access had a highly good self-perception of their physical appearance. In contrast to this study, many findings show that there is a negative relationship between access to the internet and self-perception of one's physical appearance (24-26). Many researchers show that there is a strong relationship between internet access and self-perception of one's physical (26-29). Media pressure was found to be the strongest factor enhancing internalization for adolescents. Information that is spread through mass media, especially internet-based media, is usually unrealistic, and research into body image has suggested that internalization results in a discrepancy between actual and ideal body image, leading to low body esteem (30).~~

Currently, films occupy a significant portion of the media products consumed by people (31). It is the most popular way of spending leisure time and has the greatest influence in modern life (32). Different studies have identified many positive effects of films/movies on children and adolescents; one of these is the self-concept of one’s physical appearance. This positive effect is based on the selection of movies/films that fit children and adolescent development stages (33,34). As such, the results of this study also show that there is a strong relationship at $p<0.01$ between the self-perception of one’s physical appearance of oneself and adherence/following both Ethiopian and Western movies. However, some studies show that there is a negative relationship between adherence to cinema/films and self-perception of one’s physical appearance (32,35).

5. CONCLUSION AND RECOMMENDATION

Having access to a suitable sport field is very important for children's physical development. Physical fitness is related to the ability to perform physical activity and evaluate one’s body image (23). Adolescent physical self-esteem can be improved by participating in sports. Health benefits of exercise include increased aerobic power, increased muscle strength, and reduced obesity (24,25). As such, the results of this study all support this reality. Those who reported that there was enough football field and physical activity in the school had a highly good self-perception of their physical appearance. This result is consistent with a study performed in Canada (24). Similarly, it is supported by a review paper focused on school-based physical activity, body image, and academic performance.

The findings of this study show that there is a positive relationship between using internet services and self-perception of one’s physical appearance. This means, compared to adolescents who did not have access to the internet, those who had internet access had a highly good self-perception of their physical appearance. In contrast to this study, many findings show that there is a negative relationship between access to the internet and self-perception of one’s physical appearance (26–

28). Many researchers show that there is a strong relationship between internet access and self-perception of one's physical (28–31). Media pressure has been found to be the most powerful factor driving adolescent internalization. Information disseminated through mass media, especially online media, is often unrealistic, and body image research suggests that assimilation leads to discrepancies between actual and ideal body image, leading to lower self-esteem. has been shown (32).

Movies now make up the majority of media products people consume (33). It is the most common way of spending leisure time and has the greatest impact on modern life (34). Various studies have confirmed the many positive effects of movies on children and adolescents. One of them is the self-concept of appearance. This positive effect depends on the selection of films/movies appropriate for the developmental stages of children and adolescents (35,36). As such, the results of this study also show a strong relationship, $p < 0.01$, between self-perceptions of physical appearance and adherence/following of Ethiopian and Western films. However, some studies show that there is a negative relationship between adherence to cinema/films and self-perception of one's physical appearance (34,37).

Conclusion and recommendation

According to this study, only 48.4% of ~~all~~ respondents reported a good self-perception of ~~one's~~their physical appearance. This finding indicates that adolescents need to work to strengthen their ~~physical~~good self-perception~~.~~ of their physical appearance. The results ~~of the present study~~also show ~~significant correlations between good self-perception of one's physical appearance and their~~that, respondents' BMI~~ of respondents~~, availability of enough sport fields in the school they are learning in, ~~Daily~~daily access to internet service, following Ethiopian

Formatted: Line spacing: Double

~~Movies~~movies/cinemas regularly, following ~~Western Movies/cinemas~~western music regularly~~.~~ are factors that significantly determine the good self-perception of one's physical appearance. Adolescents and their families need to work to ~~bring~~achieve normal weight and height to develop a normal body mass index, which strengthens self-perceptions of their physical appearance. Schools need to increase access to enough sport fields to strengthen their physical fitness, which increases their self-perception of their physical appearance. Additionally, adolescents need to follow internet access, media, and movies/cinema selectively as they fit their developmental stage. Future research should consider well-organized studies that include many variables that enable the determination of underlying causes of poor self-perception of ~~one~~one's physical appearance.

~~6. ABBREVIATIONS~~

Abbreviations

AOR: Adjusted Odds Ratio, BMI: Body Mass Index, CI: Confidence Interval, COR: Crude Odds Ratio, MUAC: Most Upper Arm Circumference, REC: Research and Ethical Committee, SD: Standard Deviation, SPSS: Statistical Package for Social Science

~~7. DECLARATIONS~~

Declarations

~~7.1.~~ Acknowledgement

My special thanks go to the Addis Ababa city health bureau all schools included in the study for their willingness to give me supportive letters and important information for my work. Last, I would like to thank all participants included in the study for their willingness to participate.

Formatted: Line spacing: Double

Formatted: Font: 16 pt, Font color: Auto

Formatted: Font: 16 pt, Not Bold

Formatted: Heading 2, None, Space After: 12 pt, Line spacing: single, No bullets or numbering, Don't keep with next, Don't keep lines together

Formatted: Line spacing: Double

7.2.Authors contribution

ZAA hypothesized and developed the objective, managed database development and analysis and primarily wrote the manuscript. SAS was involved in developing objective and data analysis and assisted in manuscript revision. AN assisted in data analysis and revised the manuscript. All authors read and approved the final manuscript.

7.3. Funding

No Funding The authors received. no specific funding for this work.

7.4. Availability of data and materials

The datasets used and/or analyzed during the current study are not publicly available but are available from the corresponding author on reasonable request.is uploaded as Supporting Information file.

7.5. Ethics approval and consent to participate

Before conducting the study, ethical clearance was obtained from the Addis Ababa Health Bureau ethical clearance board and Addis Ababa educational bureau. Participants' right to self-determination and autonomy was respected, and study participants were given any information they needed verbally and in written prior to self-administration. For those who were below 18 years old, consent from their parents was obtained. The rights of each respondent refusing to answer a few or all questions were respected. Participation was voluntary, and participants could withdraw from the study at any time without explanation.

7.6. Consent for publication

Not Applicable.

Formatted: Font: 16 pt, Font color: Auto

Formatted: Heading 2, Space After: 12 pt, No bullets or numbering, Adjust space between Latin and Asian text, Adjust space between Asian text and numbers

Formatted: Font: 16 pt, Not Bold

Formatted: Line spacing: Double

Formatted: Font: 13 pt, Font color: Auto

Formatted: Heading 2, Space After: 12 pt, No bullets or numbering, Adjust space between Latin and Asian text, Adjust space between Asian text and numbers

Formatted: Font: Not Bold

Formatted: Line spacing: Double

Formatted: Font: 16 pt, Font color: Auto

Formatted: Heading 2, Space After: 12 pt, No bullets or numbering

Formatted: Font: 16 pt, Not Bold

Formatted: Line spacing: Double

Formatted: Font: 16 pt, Font color: Auto

Formatted: Heading 2, Space After: 12 pt, No bullets or numbering

Formatted: Font: 16 pt, Not Bold

Formatted: Normal, Line spacing: Double, No bullets or numbering

~~7.7.~~ Competing interest

The authors declare that they have no competing interests.

~~8. REFERENCE~~

Reference

1. Riel CAHP Van, Bergh EEMM Den, Kemps HLM, Feuth T, Schreuder HWB, Hoogerbrugge PM, et al. Self-perception and quality of life in adolescents during treatment for a primary malignant bone tumour. Eur J Oncol Nurs~~.~~ [Internet]. 2014;18(3):267–72. Available ~~from~~at: http://dx.doi.org/10.1016/j.ejon.2014.01.005

2. Zadorozhnaya EG and L. Self-perception of Physical Appearance in Adolescents : Gender , Age and Self-perception of Physical Appearance in Adolescents : Gender , Age and Ethnic Aspects. Coll Antropol. 2016;40(2):73–81.

3. Vasquez P, Luarte-rocha C. Assessment of self-perception of physical fitness and proposal for standards among Chilean adolescent students : the EAPAF study. Arch Argent Pediatr. 2016;114(4):319–28.

4. Godina E, Zadorozhnaya L. Self-perception of Physical Appearance in Adolescents : Gender , Age and Ethnic Aspects. Coll Antropol. 2016;40:73–81.

5. Tlaye KG, Belete MA, Demelew TM, Getu MA. Reproductive health services utilization and its associated factors among adolescents in Debre Berhan town , Central Ethiopia : a community-based cross-sectional study. 2018;1–11.

6. Tubić T, Đorđić V. Age and gender effects on global self-worth and domain-specific self-

Formatted: Font: 16 pt, Font color: Auto

Formatted: Heading 2, Space After: 12 pt, No bullets or numbering, Adjust space between Latin and Asian text, Adjust space between Asian text and numbers

Formatted: Font: 16 pt, Not Bold

Formatted: Normal, Line spacing: Double, No bullets or numbering

Formatted: Line spacing: Double

perceptions in youth. Tatjana Tubić and Višnja Đorđić. 2015;41–61.

7. Nicolosi S, Lipoma M. ~~self~~Self-esteem, physical self-perception and physical activities programs for adolescents~~ Simona.~~. Stefan De Wals KM, redakteur. Rome: Nova Science Publishers, Inc; 2016. 299–310 bl.

~~8. Differences A. self~~8. Glauber Carvalho Nobre NCV. Self-perception of competence: concept, changes in childhood, and gender and age-group differences. J Phys Educ. 2019;30(2):1–10.

9. Santana LP, R RDC, Ana MO, Rosa M, Ester PC, Pinto DJ, et al. Factors associated with body image dissatisfaction among adolescents in public schools students in Salvador , Brazil. Nutr Hosp. 2013;28(3):747–55.

10. Al-zyoudi M. Gender differences in self-concept among adolescents with low vision. Int J Spec Educ. 2007;22(1):132–6.

11. Manning MA. Self-Concept and Self-Esteem in Adolescents~~.~~ [Internet]. Anne Arundel; 2007. Available ~~from~~at: www.naspcenter .org/principals

12. Esnaola I, Ses A. The Development of Multiple Self-Concept Dimensions During Adolescence. J Res Adolesc. 2018;30(2016):100–14.

13. Kelly LH. Florida State University Libraries The Relationship Between Physical Self-Concept , Body Image Dissatisfaction and Competition Anxiety in Female " Aesthetic " and " Non-Aesthetic " Collegiate Athletes. Florida State University; 2004.

~~14. Bisrat Kifle Arsisoa, Gizaw Mengistu Tsiduc, Gerrit Hendrik Stoffberg TT. Influence of~~

~~urbanization-driven land use/cover change on climate: The case of Addis Ababa, Ethiopia. Phys Chem Earth. 2018;105:212–23.~~

~~15~~14. Şahİn E, Barut Y, Ersanli E, Kumcağız H. Self-esteem and Social Appearance Anxiety : An Investigation of Secondary School Students. J Basic Appl Sci Res. 2014;4(3):152–9.

~~16~~15. Moehlecke M, Blume CA, Cureau FV, Kieling C, Schaan BD. Self-perceived body image, dissatisfaction with body weight and nutritional status of Brazilian adolescents: a nationwide study. J Pediatr (Rio J). 2020;96(1):76–83.

~~17~~16. da Silva AF, Moraes MS, Martins PC, Pereira EV, de Farias JM, Silva DAS. Prevalence of body image dissatisfaction and association with teasing behaviors and body weight control in adolescents. Motriz Rev Educ Fis. 2020;26(1):1–11.

~~18~~17. Ribeiro-Silva R de C, Fiaccone RL, Conceição-Machado MEP da, Ruiz AS, Barreto ML, Santana MLP. Body image dissatisfaction and dietary patterns according to nutritional status in adolescents. J Pediatr (Rio J). 2018;94(2):155–61.

~~19~~18. Weiss MR. Developmental Sport Psychology. Int Encycl Soc Behav Sci. 2001;3620–4.

19. Godina E. Self-perception of Physical Appearance in Adolescents : Gender , Age and Self-perception of Physical Appearance in Adolescents : Gender , Age and Ethnic Aspects. Coll Antropol. 2016;40(2):73–81.

20. Addis Ababa City adminstration Human resource management and development Bureau Annual report. 2019.

~~20~~21. Cole TJ, Flegal KM, Nicholls D, Jackson AA. Body mass index cut offs to define thinness in children and adolescents: International survey. Br Med J. 2007;335(7612):194–7.

Formatted: Line spacing: Double

Formatted: Line spacing: Double

22. Pop CL. Association between body mass index and self body image perception. Iran J Public Health. 2017;46(12):1744–5.

~~21~~23. Balsalobre FJB, Sánchez GFL, Suárez AD. Relationships between Physical Fitness and Physical Self-concept in Spanish Adolescents. Procedia - Soc Behav Sci [Internet]. 2014;132:343–50. Available ~~from~~at: http://dx.doi.org/10.1016/j.sbspro.2014.04.320

~~22~~24. Bowker A. The relationship between sports participation and self-esteem during early adolescence. Can J Behav Sci. 2006;38(3):214–29.

~~23~~25. Organized Sports for Children and Preadolescents. Pediatrics. 2001;107(6):1459–62.

~~24~~26. Rohall DE, Cotten SR, Morgan C. Internet use and the self concept: Linking specific uses to global self-esteem. Curr Res Soc Psychol. 2002;8(1):1–18.

~~25~~27. Hogan SR. The Effect of Social Media on Body Image Among Adolescents Results Methods. Calif State Univ. 2017;46(6).

~~26~~28. Rodgers RF, Melioli T. The Relationship Between Body Image Concerns, Eating Disorders and Internet Use, Part I: A Review of Empirical Support. Adolesc Res Rev. 2016;1(2):95–119.

~~27~~29. Hogue J V., Mills JS. The effects of active social media engagement with peers on body image in young women. Body Image [Internet]. 2019;28:1–5. Available ~~from~~at: https://doi.org/10.1016/j.bodyim.2018.11.002

~~28~~30. Brown T, Champ J. Examining the Influence of Social Media on Body Image: Miss Perfection, a Misperception [Internet]. ProQuest Dissertations and Theses. Colorado State

Formatted: Line spacing: Double

University; 2017. Available ~~from~~at: https://mountainscholar.org/handle/10217/183897

~~29~~31. Lee HR, Lee HE, Choi J, Kim JH, Han HL. Social media use, body image, and psychological well-being: A cross-cultural comparison of korea and the united states. J Health Commun. 2014;19(12):1343–58.

~~30~~32. You S. Body Esteem among Korean Adolescent Boys and Girls. Sustainability. 2019;11(2051):1–13.

~~31~~33. Kubrak T. Impact of films: Changes in young People's attitudes after watching a movie. Behav Sci (Basel). 2020;10(5).

~~32~~34. Mahmood I. Influence and Importance of Cinema on the Lifestyle of Educated Youth: A study on University Students of Bangladesh. IOSR J Humanit Soc Sci. 2013;17(6):77–80.

~~33~~35. Javellana GM. Influence of Media on Body Image Satisfaction among Adolescents. Asia Pacific J Educ Arts Sci | [Internet]. 2014;1(1):95–101. Available ~~from~~at: http://apjeas.apjmr.com/wp-content/uploads/2014/04/APJEAS-2014-1-024.pdf

~~34~~36. Agarwal V, Dhanasekaran S. Harmful effects of media on children and adolescents. J Indian Assoc Child Adolesc Ment Heal. 2012;8(2):38–45.

~~35~~37. Hefner V, Woodward K, Figge L, Bevan JL, Santora N, Baloch S. The Influence of Television and Film Viewing on Midlife Women's Body Image, Disordered Eating, and Food Choice. Media Psychol. 2014;17(2):185–207.

Dear Editor,

Thank you for giving us the opportunity to submit a revised draft of our manuscript titled (Self-perception of physical appearance of adolescents and associated factors in Addis Ababa, Ethiopia) to *Plos One journal*. We appreciate you and the reviewer for your precious time in reviewing our paper and providing valuable suggestion. It was your valuable and insightful comments that led to possible improvements in the current version. The authors have carefully considered the comments and tried our best to address every one of them. We hope the manuscript after careful revisions meet your high standards. The authors welcome further constructive comments if any.

Below we have provide the point-by-point responses/clarification to editor and reviewer comments. In addition, all modifications in the manuscript have been highlighted in red in document uploaded named as "Revised Manuscript with Track Changes".

1. **Comments from Editor:**

Comment 1: Please ensure that your manuscript meets PLOS ONE's style requirements, including those for file naming.

Response: We had revised the manuscript as per PLOS ONE's style requirements including the file naming.

Comment 2: Statement related to financial disclosure.

Response: The authors received no specific funding for this work. The same statement is included in the manuscript.

Comment 3: Issue related to data availability.

Response: We have uploaded the underlying data as supporting Information files

Comment 4: Your ethics statement should only appear in the Methods section of your manuscript.

Response: The ethical statement is included under method section by removing from declaration part.

Comment 5: Thank you for submitting the above manuscript to PLOS ONE. During our internal evaluation of the manuscript, we found significant text overlap between your submission and the following previously published works, some of which you are an author.

Response: We have made great effort to replace, Re-write and adjust those overlapping text.

2. **Comments from the reviewer:**

Abstract section comments:

Comment 1: Introduction: The clear research gap is not clearly stated. The researcher describes the importance of positive body image for physical and mental health. However, the research what is unknown and what the current research adds a new knowledge are not well described.

I would suggest remove the highlighted words from the aim of the study "Therefore, the aim of this study was to assess the level of self-perception of physical appearance and its associated factors among adolescents in Addis Ababa, Ethiopia."

Response: Thank you for your valuable comment. We have tried to show the research gap and what the current research add to current knowledge. Moreover, as abstract word count is limited it is impossible to explain more in this part, so that we had tried to explained more in the introduction part.

Comment 2: The researcher did not describe how the multistage sampling was done. Its needs a summary of report for the multistage sampling in abstract section because the reader does not understand the sampling units were schools, or districts, or others. It is also better to describe what variables are measured and how before they tell us the analysis. For example, self-perception is measured using structured questionnaire (it is good to mention whether it is validated tool or tools were developed for this study) and describe how the data was collected such as using 'self-administered' or 'interview'. Some other detail information can be presented in the methods section of the manuscript. I also suggest to define the self-perception classification if it has. For example, the researcher said the "over all good self-perception", what does it mean? The researcher also reported there was also said the good self-perception was low in the conclusion section which need definition in the method section. Indeed, summary of the important methods section needs further improvement.

Response: Thank you again for the comment: The summary of report for the multistage sampling is included in the abstract part as per your comment.

The variables measured are included in the manuscript under method section subsection study variables.

The dependent variable are defined under methodology section

We had tried to modify the methodology part to make it clearer to the reader by incorporating all your comments. Please see the methodology section of the manuscript. As you know, under abstract section it is difficult to explain every points as this part word count allowed is very limited as per the journal format.

Comment 3: Having BMI between 18.5 kg/m2-24.9 kg/m2 (AOR=2.56; 95% CI: 1.45, 4.54), presence of enough sport fields in the school (AOR=1.89; 95% CI: 1.09, 3.29), having daily access to internet services (AOR=1.69; 95% CI: 1.07, 2.94), following Ethiopian movies/cinemas (AOR=2.46; 95% CI: 1.46, 4.15), and regularly following Western movies/cinemas (AOR=2.0; 95% CI: 1.11, 3.59) were significantly associated with self-perception of one's physical appearance

Does it associated with …good self-perception low self-perception? Make it clear or you may say "had statistically significant positive association with self-perception of one's appearance

Response: The comment is well received and corrected accordingly in the manuscript.

Comment 4: Conclusions: the researcher need to know the nature of a cross-sectional study to determine the associated factors. Thus, researcher must be curious on the factors associated with self-perception which does not mean a causes. There might be various factors that may also associated with self-perception, for example the economy, the social factors, psychological factors, and other factors. The researcher made a shallow recommendation on how the current knowledge is used.

The researcher recommends to increase the access to sport fields …not clear. This look a very general recommendation one can do without

Key words ….>keywords

Response: Of course there might be many factors that associated with the self-perception of one's physical appearance other than that discussed here. However, we explained and discussed the factors depending the result of our analysis. In addition, as you know the recommendation must be depend on the finding of the study. Anyhow we have adjusted the recommendation to some extent.

It is not clear about comment of “Key words” We have included the possible keywords in the manuscript.

Introduction section comments

Comment 5: It would be good to include a paragraph explaining how self-perception may be influenced by a variety of factors.

It is inconsistent throughout the document to use terms like 'dissatisfaction' and 'low self-perception'.

Responses: We have included a paragraph explaining how varies factors do affect self-perception of physical appearance.

In relation to 'dissatisfaction' and 'low self-perception', some literatures use the first, other use the second one. Hence, in the introduction section part of this manuscript, there may be either of them or both because more of the introduction part was extracted from previous studies that might use either of them to explain all most the same issue. However, the finding of this study is depend on the operational definition we had provided in the methodology part which is either Good self-perception or Poor self-perception.

Comment 6: The study was a community based cross sectional study. Why the study include only adolescent who attend high school education? Would not be easy to make institution (based) cross-sectional study if the participants are only adolescent who attend high school education?

Response: To make this point clear, this study was first planned to be done at institutional (school) level, however, due to covid-19, the schools were closed as part of covid-19 prevention protocol. Then we have changed to community based. So, the sampling unit is household not school.

Comment 7: The researcher selects two sub-cities from Addis Ababa, Ethiopia from 10 or now is 11 sub-cities. Do you think that the result of the study generalizable to Addis Ababa?

Response: At the time of starting of this work, the sub cities in Addis Ababa were 10. Hence, we have selected only two sub cities due to limitation of available resources. However, as much as possible, we tried to make the sampling representative by using a simple random sampling technique to select the sub cities and districts.

Comment 8: I also highly recommend a thorough formatting of the paper for publication in the journal.

Response: We have tried to format the paper according to the journal formatting style.

Comment 9: The researcher did not mention how the two sub-cities selected? Was it randomly or purposively?

Response: The two sub cities was selected by simple random sampling system.

Comment 10: Is the sampling unit was household or school? This is not clear for the reader

Response: The sampling unit was household. This study was first planned to be done at institutional (school) level, however, due to covid-19, the schools were closed as part of covid-19 prevention protocol. Then we have changed to community based. So, the sampling unit is household not school.

Comment 11: How many household or school was included in the study?

Response: 308 households was included**.**

Comment 12: It is also not clear how simple random sampling was applied at community based cross-sectional study or was it school based

Response: The comment is well taken. It is typing error. Corrected to Systematic random sampling system.

Comment 13: The sample size calculation not clearly described. What was the proportion of 19.5%? Was it dissatisfaction on self-perception? Why the researcher uses the proportion 50% which can give the maximum sample size for the cross-sectional study. The researcher also did not describe the adjustment of sample size because of multistage sampling strategy.

Response. Corrected and we had included sample size calculation description. 19.5% is the proportion of satisfaction not dissatisfaction. As you know 50% is recommended if there is no any previous study done on the topic. Thus why we used the available reference.

Comment 13: Dependent Variable: Level of self-perception of physical appearance: bring the operational definition here.

Response: We had already included operational definition in methodology part. However, as per your comment we have merged with dependent variable.

Comment 14: The researcher is not consistently used terms in relation to self-perception. In the introduction section they used terms ‘dissatisfaction’ and in the method they used ‘good-self-perception’ and ‘low-self-perception’.

Response: In relation to 'dissatisfaction' and 'low self-perception', some literatures use the first, other use the second one. Hence, in the introduction section part of this manuscript, there may be either of them or both because more of the introduction part was extracted from previous studies that might use either of them to explain all most the same issue. However, the finding of this study is depend on the operational definition we had provided in operational definition in the methodology part which is either Good self-perception or Poor self-perception.

Comment 15: Why the researcher wants to use the mean score to classify the self-perception as mean can be affected by skewness. Did the researcher checked the normal distribution of the data before they decide to use the mean score classification?

Responses: We used mean as cut-point, because our data is normally distributed data.

Comment 16: The classification term also makes confuse the reader, for example, in the abstract section the researchers said, “Good perception of one’s physical appearance was low”. This makes confused with the self-perception classification ‘’Good” and “low”. I would suggest “good” and “poor’ or “high” and “low” or “satisfied” and “dissatisfied”

Responses: It good and appropriate comment, so, as per your comment we corrected the operational definition as high” and “poor” satisfaction. As such, this operational definition was used to describe the satisfaction level of the finding of the study.

Comment 17: I would suggest making the operational definition together with the dependent variable.

Response: Comment total accepted, the operational definition merged with dependent variable.

Comment 18: The researcher did not clearly describe the variables which needs revision

Response: Comment accepted and revised accordingly.

Comment 19: The researchers did not clearly describe the measurements in details. For examples to measure the self-perception of physical how many questions were used and on what type? Was it liker or yes/No answer options.

Response: Comment total accepted and corrected accordingly. See Methodology part sub section Data collection tools and quality control which explain the measurments.

Comment 20: There is a repetition of statement

Prior to the actual data collection, the questionnaire was adjusted and 18 corrected based on the pretest result, and the final questionnaire was translated to Amharic and 19 then back to English to ensure consistency. "The questionnaire was pretested and modified 25 based on the findings related to clarity, wordings, logical sequence, skip patterns of the questions 26 and resources needed."

Merge these two statements into one

Response: Comment Accepted. Merged accordingly.

Comment 21: The researcher did not explained why BMI and MUAC measurement were used together? Study showed BMI and MUAC highly correlated in Adolescent. What was the assumptions behind using this two indices for nutritional assessment?

Responses: The comment is right, different literatures showed that BMI and MUAC measurements are highly correlated. We assumed that the findings of our study may show the research communities another support in addition to available evidences if both measurements are done simultaneously. Besides, the assumption behind using these two indices for nutritional assessment among adolescents proving researchers thoughts such as "The MUAC is useful especially in monitoring severe under nutrition" and "The BMI is a commonly used measurement for screening both underweight and overweight".

Comment 22: Were the researchers considering any assumptions for the appropriateness of the analysis they used? Why logistics regression analysis? Did the researcher check the assumption for it?

Response: As our data is categorical data, we used logistics regression analysis which does not require linearity test. We have made same assumptions before starting the analysis. Example we have checked multi-collinearity test and our data is free from it.

Comment 23: According to WHO BMI-for age (Obese: >2SD; Overweight: >1SD; Normal: 1 - -2SD; Thinness: <− 2 SD; Severe thinness: <− 3 SD) and height-for-age below -2SD is used for stunting. The MUAC for age can also be used classified for adolescent

age. The researcher did not clearly describe the BMI and MUAC classification in this regard. This needs to be clearly stated with the reference. This need to be clearly stated with the reference.

Response: We included the BMI classification explanation with reference in the methodology part according to your comment. Please see sub section data collection tools and quality control under methodology part.

Result section comments

Comment 24: What was the base for monthly income classification?

Responses: The classification of income is depend on the mean value of the income level taken as cut-off point.

Comment 25: According to responses rated based on five Likert scales, namely, 1-strongly disagree, 2- 4 disagree, 3-not sure, 4-agree and 5-strongly agree, the overall mean perception of respondents 5 was 2.9 (±0.02). Based on the definition of good and low perception, approximately 157 (55.5%) 6 respondents had good perception, and 126 (44.6%) had low perception.

The above statement is not clear for reader

Response: To make it more clear, we re-write it and add a table
