# Supplementary material for: Self-perception of physical appearance of adolescents and associated factors in Addis Ababa, Ethiopia
Source: PLoS One. 2023 Jan 27;18(1):e0281202. doi: 10.1371/journal.pone.0281202 (PMC9882736; doi:10.1371/journal.pone.0281202)
Supplement: Attachment — Submitted filename: Reviewers comment - PONE-D-22-00860.docx [file pone.0281202.s002.docx]

**Review manuscript**

**Research title**: Self-perception of physical appearance of adolescents and associated factors in Addis Ababa, Ethiopia

General comment

This researcher was selected for a very interesting title that is not well studied in developing countries. However, the study was not methodologically sound and may not be reproducible. It is necessary to improve the way scientific writing is done in the document. It is necessary to revise the research manuscript extensively

**Abstract**

**The clear research gap is not clearly stated in the introduction.** In the study, the researcher discusses the importance of positive body image for physical and mental health. Nevertheless, it is not well described what is unknown and what the current research adds to our knowledge.

I would suggest removing the highlighted words from the aim of the study “Therefore, the aim of this study was to assess the level of self-perception of physical appearance and its associated factors among adolescents in Addis Ababa, Ethiopia.”

**Method and material**

The researcher did not explain how the multistage sampling was conducted. There is a need for a summary of the report for the multistage sampling in the abstract section, as the reader does not know if the sampling units were schools, districts, or others.  It is also better to describe what variables are measured and how they are calculated before explaining the results. A structured questionnaire is used to measure self-perception (it is good to mention whether it is a validated tool or if tools were developed specifically for this study) and describe how the data was collected, such as using 'self-administered' or 'interview'. A detailed description of the methods can be found in the manuscript's methods section. If there is a self-perception classification, it should also be defined. As an example, the researcher said "overall good self-perception", what does that mean?  In the conclusion section, the researcher also reported that the good self-perception was low, which needs to be defined in the method section. The summary of the important methods section needs to be improved.

**Results:**

Having BMI between 18.5 kg/m2-24.9 kg/m2 (AOR=2.56; 95% CI: 1.45, 4.54), presence of enough sport fields in the school (AOR=1.89; 95% CI: 1.09, 3.29), having daily access to internet services (AOR=1.69; 95% CI: 1.07, 2.94), following Ethiopian movies/cinemas (AOR=2.46; 95% CI: 1.46, 4.15), and regularly following Western movies/cinemas (AOR=2.0; 95% CI: 1.11, 3.59) were significantly associated with self-perception of one’s physical appearance

What is the association between it and ...good self-perception and low self-perception? make it clear or you may say “had statistically significant positive association with self-perception of one’s appearance’

**Conclusions**:

Cross-sectional studies have limitations in determining the associated factors because they are not strong. So, the researcher needs to be curious about factors associated with self-perception, which does not mean a cause. Self-perception may also be affected by a variety of factors, such as the economy, social factors, psychological factors, etc. In terms of how the current knowledge should be applied, the researcher made shallow recommendations.

Researchers recommend increasing access to sport fields...which is not clear. This looks like a very general recommendation one can say without the current study.

Key words ….>keywords

**Introduction**

It would be good to include a paragraph explaining how self-perception may be influenced by a variety of factors.

It is inconsistent throughout the document to use terms like 'dissatisfaction' and 'low self-perception'.

**Methodology**

This study was a cross-sectional study that was conducted in a community setting. What is the purpose of including only adolescents who attend high school education in the study? Is it not possible to make an institution-based cross-sectional study if participants are only high school students?

Researchers select two sub-cities from Addis Ababa, Ethiopia out of 10 or now 11 sub-cities. Do you think that the result of the study is generalizable to Addis Ababa?

The researcher did not mention how the two sub-cities were selected? Was it randomly or purposively selected?

Was the sampling unit a household or a school? This is not clear for the reader

How many households or schools were included in the study?

It is also not clear how simple random sampling was applied while using community based cross-sectional study? The researchers did not tell us the sampling frame to use simple random sampling.

**Sample size**

There is no clear explanation of how sample size is calculated. Could you please tell me what the proportion of 19.5% was? Was it dissatisfaction with self-perception? The researcher also did not tell us if sample size is adjusted to increase because of multistage sampling strategy. Can you tell us  how many samples were calculated in total?

Why does not the researcher use the proportion 50% which will give the maximum sample size for the cross-sectional study.

**Variables**:

Dependent Variable: Level of self-perception of physical appearance: bring the operational definition here

Self-perception terms are not consistently used by the researcher. The introduction section used the term 'dissatisfaction', and the method section used the terms 'good self-perception' and 'low self-perception'.

In spite of the fact that the mean score is affected by skewness, the researcher did not explain why the mean score should be used to classify self-perception. Prior to using the mean score classification, did the researcher check the normal distribution of the data?

The classification term also confuses the reader, for example, in the abstract section, the researchers say, "good perception of one's physical appearance is low" which makes unclear for the reader. I would suggest “good” and “poor’ or “high” and “low” or “satisfied” and “dissatisfied” and define it with reference.

I would suggest making the operational definition together with the dependent variable. Thus, summarize the variables together  rather than listing. The variables description needs revision

**Data collection tools and quality**

The source of the tools is not cited. Measurements were not described in detail by the researchers. In order to measure self-perception of physical attributes, how many questions were asked and on what types of questions? Was it likert scale or binary answer yes/No options

There is a repetition of statement that needs merged and rephrase

Prior to the actual data collection, the questionnaire was adjusted and 18 corrected based on the pretest result, and the final questionnaire was translated to Amharic and 19 then back to English to ensure consistency.

 “The questionnaire was pretested and modified 25 based on the findings related to clarity, wordings, logical sequence, skip patterns of the questions 26 and resources needed.”

Merge these two statements into one

The researcher did not explain why BMI and MUAC measurements were used together. An Adolescent study found a strong correlation between BMI and MUAC. In assessing nutritional status, what assumptions were made? And were there any different things found in the two measurements ?

**Data analysis**

Were the researchers considering any assumptions for the appropriateness of the analysis they used? Why logistics regression analysis? Did the researcher check the assumption for it?

According to WHO BMI-for age (Obese: >2SD; Overweight: >1SD; Normal: 1 - -2SD; Thinness: <− 2 SD; Severe thinness: <− 3 SD) and height-for-age below -2SD is used for stunting. The MUAC for age can also be classified for adolescent age. The researcher did not clearly describe the BMI and MUAC classification in this regard. This needs to be clearly stated with the reference. The researcher classified the BMI and MUAC for adults not for adolescents. The researcher need be justified this with appropriate reference.

**Result**

**Scio-demographic characteristics**

What was the base for monthly income classification?

3.3. Perception of dietary and related condition of the respondents

 According to responses rated based on five Likert scales, namely, 1-strongly disagree, 2- 4 disagree, 3-not sure, 4-agree and 5-strongly agree, the overall mean perception of respondents 5 was 2.9 (±0.02). Based on the definition of good and low perception, approximately 157 (55.5%) 6 respondents had good perception, and 126 (44.6%) had low perception.

This statement is not clear
