# Supplementary material for: Self-perception of physical appearance of adolescents and associated factors in Addis Ababa, Ethiopia
Source: PLoS One. 2023 Jan 27;18(1):e0281202. doi: 10.1371/journal.pone.0281202 (PMC9882736; doi:10.1371/journal.pone.0281202)
Supplement: Attachment — Submitted filename: Response to Reviewers and editors comment.docx [file pone.0281202.s003.docx]

**Dear Editor,**

Thank you for giving us the opportunity to submit a revised draft of our manuscript titled (Self-perception of physical appearance of adolescents and associated factors in Addis Ababa, Ethiopia) to *Plos One journal*. We appreciate you and the reviewer for your precious time in reviewing our paper and providing valuable suggestion. It was your valuable and insightful comments that led to possible improvements in the current version. The authors have carefully considered the comments and tried our best to address every one of them. We hope the manuscript after careful revisions meet your high standards. The authors welcome further constructive comments if any.

Below we have provide the point-by-point responses/clarification to editor and reviewer comments. In addition, all modifications in the manuscript have been highlighted in red in document uploaded named as “Revised Manuscript with Track Changes”.

1. **Comments from Editor:**

**Comment 1:** Please ensure that your manuscript meets PLOS ONE's style requirements, including those for file naming.

**Response:** We had revised the manuscript as per PLOS ONE’s style requirements including the file naming.

**Comment 2:** Statement related to financial disclosure.

**Response:** The authors received no specific funding for this work. The same statement is included in the manuscript.

**Comment 3:** Issue related to data availability.

**Response:** We have uploaded the underlying data as supporting Information files

**Comment 4:** Your ethics statement should only appear in the Methods section of your manuscript.

**Response:** The ethical statement is included under method section by removing from declaration part.

**Comment 5:** Thank you for submitting the above manuscript to PLOS ONE. During our internal evaluation of the manuscript, we found significant text overlap between your submission and the following previously published works, some of which you are an author.

**Response:** We have made great effort to replace, Re-write and adjust those overlapping text.

2. **Comments from the reviewer:**

***Abstract section comments:***

**Comment 1:** Introduction: The clear research gap is not clearly stated. The researcher describes the importance of positive body image for physical and mental health. However, the research what is unknown and what the current research adds a new knowledge are not well described.

I would suggest remove the highlighted words from the aim of the study “Therefore, the aim of this study was to assess the level of self-perception of physical appearance and its associated factors among adolescents in Addis Ababa, Ethiopia.”

**Response:** Thank you for your valuable comment. We have tried to show the research gap and what the current research add to current knowledge. Moreover, as abstract word count is limited it is impossible to explain more in this part, so that we had tried to explained more in the introduction part.

**Comment 2**: The researcher did not describe how the multistage sampling was done. Its needs a summary of report for the multistage sampling in abstract section because the reader does not understand the sampling units were schools, or districts, or others. It is also better to describe what variables are measured and how before they tell us the analysis. For example, self-perception is measured using structured questionnaire (it is good to mention whether it is validated tool or tools were developed for this study) and describe how the data was collected such as using ‘self-administered’ or ‘interview’. Some other detail information can be presented in the methods section of the manuscript. I also suggest to define the self-perception classification if it has. For example, the researcher said the “over all good self-perception”, what does it mean? The researcher also reported there was also said the good self-perception was low in the conclusion section which need definition in the method section. Indeed, summary of the important methods section needs further improvement.

**Response:** Thank you again for the comment: The summary of report for the multistage sampling is included in the abstract part as per your comment.

The variables measured are included in the manuscript under method section subsection study variables.

The dependent variable are defined under methodology section

We had tried to modify the methodology part to make it clearer to the reader by incorporating all your comments. Please see the methodology section of the manuscript. As you know, under abstract section it is difficult to explain every points as this part word count allowed is very limited as per the journal format.

**Comment 3:** Having BMI between 18.5 kg/m2-24.9 kg/m2 (AOR=2.56; 95% CI: 1.45, 4.54), presence of enough sport fields in the school (AOR=1.89; 95% CI: 1.09, 3.29), having daily access to internet services (AOR=1.69; 95% CI: 1.07, 2.94), following Ethiopian movies/cinemas (AOR=2.46; 95% CI: 1.46, 4.15), and regularly following Western movies/cinemas (AOR=2.0; 95% CI: 1.11, 3.59) were significantly associated with self-perception of one’s physical appearance

Does it associated with …good self-perception low self-perception? Make it clear or you may say “had statistically significant positive association with self-perception of one’s appearance

**Response:** The comment is well received and corrected accordingly in the manuscript.

**Comment 4:** Conclusions: the researcher need to know the nature of a cross-sectional study to determine the associated factors. Thus, researcher must be curious on the factors associated with self-perception which does not mean a causes. There might be various factors that may also associated with self-perception, for example the economy, the social factors, psychological factors, and other factors. The researcher made a shallow recommendation on how the current knowledge is used.

The researcher recommends to increase the access to sport fields …not clear. This look a very general recommendation one can do without

Key words ….>keywords

**Response:** Of course there might be many factors that associated with the self-perception of one’s physical appearance other than that discussed here. However, we explained and discussed the factors depending the result of our analysis. In addition, as you know the recommendation must be depend on the finding of the study. Anyhow we have adjusted the recommendation to some extent.

It is not clear about comment of “Key words” We have included the possible keywords in the manuscript.

***Introduction section comments***

**Comment 5:** It would be good to include a paragraph explaining how self-perception may be influenced by a variety of factors.

It is inconsistent throughout the document to use terms like 'dissatisfaction' and 'low self-perception'.

**Responses:** We have included a paragraph explaining how varies factors do affect self-perception of physical appearance.

In relation to 'dissatisfaction' and 'low self-perception', some literatures use the first, other use the second one. Hence, in the introduction section part of this manuscript, there may be either of them or both because more of the introduction part was extracted from previous studies that might use either of them to explain all most the same issue. However, the finding of this study is depend on the operational definition we had provided in the methodology part which is either Good self-perception or Poor self-perception.

**Comment 6:** The study was a community based cross sectional study. Why the study include only adolescent who attend high school education? Would not be easy to make institution (based) cross-sectional study if the participants are only adolescent who attend high school education?

**Response:** To make this point clear, this study was first planned to be done at institutional (school) level, however, due to covid-19, the schools were closed as part of covid-19 prevention protocol. Then we have changed to community based. So, the sampling unit is household not school.

**Comment 7:** The researcher selects two sub-cities from Addis Ababa, Ethiopia from 10 or now is 11 sub-cities. Do you think that the result of the study generalizable to Addis Ababa?

**Response:** At the time of starting of this work, the sub cities in Addis Ababa were 10. Hence, we have selected only two sub cities due to limitation of available resources. However, as much as possible, we tried to make the sampling representative by using a simple random sampling technique to select the sub cities and districts.

**Comment 8:** I also highly recommend a thorough formatting of the paper for publication in the journal.

**Response:** We have tried to format the paper according to the journal formatting style.

**Comment 9:** The researcher did not mention how the two sub-cities selected? Was it randomly or purposively?

**Response**: The two sub cities was selected by simple random sampling system.

**Comment 10:** Is the sampling unit was household or school? This is not clear for the reader

**Response:** The sampling unit was household. This study was first planned to be done at institutional (school) level, however, due to covid-19, the schools were closed as part of covid-19 prevention protocol. Then we have changed to community based. So, the sampling unit is household not school.

**Comment 11:** How many household or school was included in the study?

**Response:** 308 households was included**.**

**Comment 12:** It is also not clear how simple random sampling was applied at community based cross-sectional study or was it school based

**Response:** The comment is well taken. It is typing error. Corrected to Systematic random sampling system.

**Comment 13:** The sample size calculation not clearly described. What was the proportion of 19.5%? Was it dissatisfaction on self-perception? Why the researcher uses the proportion 50% which can give the maximum sample size for the cross-sectional study. The researcher also did not describe the adjustment of sample size because of multistage sampling strategy.

**Response.** Corrected and we had included sample size calculation description. 19.5% is the proportion of satisfaction not dissatisfaction. As you know 50% is recommended if there is no any previous study done on the topic. Thus why we used the available reference.

**Comment 13:** Dependent Variable: Level of self-perception of physical appearance: bring the operational definition here.

**Response:** We had already included operational definition in methodology part. However, as per your comment we have merged with dependent variable.

**Comment** **14:** The researcher is not consistently used terms in relation to self-perception. In the introduction section they used terms ‘dissatisfaction’ and in the method they used ‘good-self-perception’ and ‘low-self-perception’.

**Response:** In relation to 'dissatisfaction' and 'low self-perception', some literatures use the first, other use the second one. Hence, in the introduction section part of this manuscript, there may be either of them or both because more of the introduction part was extracted from previous studies that might use either of them to explain all most the same issue. However, the finding of this study is depend on the operational definition we had provided in operational definition in the methodology part which is either Good self-perception or Poor self-perception.

**Comment 15:** Why the researcher wants to use the mean score to classify the self-perception as mean can be affected by skewness. Did the researcher checked the normal distribution of the data before they decide to use the mean score classification?

**Responses:** We used mean as cut-point, because our data is normally distributed data.

**Comment 16:** The classification term also makes confuse the reader, for example, in the abstract section the researchers said, “Good perception of one’s physical appearance was low”. This makes confused with the self-perception classification ‘’Good” and “low”. I would suggest “good” and “poor’ or “high” and “low” or “satisfied” and “dissatisfied”

**Responses:** It good and appropriate comment, so, as per your comment we corrected the operational definition as high” and “poor” satisfaction. As such, this operational definition was used to describe the satisfaction level of the finding of the study.

**Comment 17:** I would suggest making the operational definition together with the dependent variable.

**Response:** Comment total accepted, the operational definition merged with dependent variable.

**Comment 18:** The researcher did not clearly describe the variables which needs revision

**Response:** Comment accepted and revised accordingly.

**Comment 19:** The researchers did not clearly describe the measurements in details. For examples to measure the self-perception of physical how many questions were used and on what type? Was it liker or yes/No answer options.

**Response:** Comment total accepted and corrected accordingly. See Methodology part sub section Data collection tools and quality control which explain the measurments.

**Comment 20:** There is a repetition of statement

Prior to the actual data collection, the questionnaire was adjusted and 18 corrected based on the pretest result, and the final questionnaire was translated to Amharic and 19 then back to English to ensure consistency.
“The questionnaire was pretested and modified 25 based on the findings related to clarity, wordings, logical sequence, skip patterns of the questions 26 and resources needed.”
Merge these two statements into one

**Response:** Comment Accepted. Merged accordingly.

**Comment 21:** The researcher did not explained why BMI and MUAC measurement were used together? Study showed BMI and MUAC highly correlated in Adolescent. What was the assumptions behind using this two indices for nutritional assessment?

**Responses:** The comment is right, different literatures showed that BMI and MUAC measurements are highly correlated. We assumed that the findings of our study may show the research communities another support in addition to available evidences if both measurements are done simultaneously. Besides, the assumption behind using these two indices for nutritional assessment among adolescents proving researchers thoughts such as “The MUAC is useful especially in monitoring severe under nutrition” and “The BMI is a commonly used measurement for screening both underweight and overweight”.

**Comment 22:** Were the researchers considering any assumptions for the appropriateness of the analysis they used? Why logistics regression analysis? Did the researcher check the assumption for it?

**Response:** As our data is categorical data, we used logistics regression analysis which does not require linearity test. We have made same assumptions before starting the analysis. Example we have checked multi-collinearity test and our data is free from it.

**Comment 23:** According to WHO BMI-for age (Obese: >2SD; Overweight: >1SD; Normal: 1 - -2SD; Thinness: <− 2 SD; Severe thinness: <− 3 SD) and height-for-age below -2SD is used for stunting. The MUAC for age can also be used classified for adolescent age. The researcher did not clearly describe the BMI and MUAC classification in this regard. This needs to be clearly stated with the reference. This need to be clearly stated with the reference.

**Response:** We included the BMI classification explanation with reference in the methodology part according to your comment. Please see sub section data collection tools and quality control under methodology part.

***Result section comments***

**Comment 24:** What was the base for monthly income classification?

**Responses:** The classification of income is depend on the mean value of the income level taken as cut-off point.

**Comment 25:** According to responses rated based on five Likert scales, namely, 1-strongly disagree, 2- 4 disagree, 3-not sure, 4-agree and 5-strongly agree, the overall mean perception of respondents 5 was 2.9 (±0.02). Based on the definition of good and low perception, approximately 157 (55.5%) 6 respondents had good perception, and 126 (44.6%) had low perception.

The above statement is not clear for reader

**Response:** To make it more clear, we re-write it and add a table
