# Supplementary material for: Self-perception of physical appearance of adolescents and associated factors in Addis Ababa, Ethiopia
Source: PLoS One. 2023 Jan 27;18(1):e0281202. doi: 10.1371/journal.pone.0281202 (PMC9882736; doi:10.1371/journal.pone.0281202)
Supplement: Attachment — Submitted filename: Response to Reviewers and editors comment.docx [file pone.0281202.s005.docx]

**Dear Editor,**

Thank you for giving us the opportunity to submit a revised draft of our manuscript titled (Self-perception of physical appearance of adolescents and associated factors in Addis Ababa, Ethiopia) to *Plos One journal*. We appreciate you and the reviewer for your precious time in reviewing our paper and providing valuable suggestion. It was your valuable and insightful comments that led to possible improvements in the current version. The authors have carefully considered the comments and tried our best to address every one of them. We hope the manuscript after careful revisions meet your high standards. The authors welcome further constructive comments if any.

Below we have provide the point-by-point responses/clarification to editor and reviewer comments. In addition, all modifications in the manuscript have been highlighted in red in document uploaded named as “Revised Manuscript with Track Changes”.

1. **Comments from Academic Editor:**

**Comment 1:** In the abstract section please rephrase this statement "Thus, the aim of study was to generate evidence by assessing self-perception of physical appearance and its associated factors among adolescents in Addis Ababa." as "Thus, the aim of this study was to assess the self-perception of physical appearance and its associated factors among adolescents in Addis Ababa, Ethiopia."

**Response:** The comment is well accepted and corrected accordingly.

**Comment 2:** Please check the English language throughout the manuscript document

**Response:** The authors received no specific funding for this work. The same statement is included in the manuscript.

2. **Comments from the reviewer:**

***Abstract section comments:***

**Comment 1: *Methods and Materials:*** (Logistic regression) need paraphrasing to make it more clearly for the readers. Write it in detail because some readers only read the abstract.

**Response:** Thank you for your valuable comment. We have tried to make clearer by adding some explanation.

**Comment 2:** ***Result:*** Add response rate

**Response:** The comment is well accepted and added accordingly.

**Comment 3:** **Conclusion:** Compared to Local findings or from global studies. Such conclusion need careful overview.

**Response:** We have tried to conclude according to your suggestion.

**Comment 4:** **Conclusion:** Schools and the local administrations need to increase access to enough sport fields for students to strengthen their physical fitness, which increases their good self-perception of their physical appearance. (**How this recommendation is drawn from the result is not clear).**

**Response:** The recommendation is drown because the availability and accessibility of enough sport fields in the school was one of the determinant factor for good self-perception of one’s physical appearance according to the finding of this study.

***Introduction section comments:***

- All specific comments under introduction parts were well addressed and the changes done is highlighted in the document with track change.

***Method and Material section Comments:***

**Comment 1:** Study setting and design: In what year does the specified number of Addis Ababa population.

**Response:** The comment is corrected by including the current (2022) population size of Addis Ababa.

**Comment 2:** Since you have used a multi-stage sampling it is expected to consider design effect. If it is not possible to a correct this at this stage, I suggest you can modify the study population, otherwise with sampling it is difficult to generalize.

**Response:** We consider the population as homogeneous. So that, using the calculated sample size without adding design effect may not significantly affect the generalizability of the result. Also due to resource constraint we decided to conduct with this sample size.

**Comment 3:** Do you have any reference to use mean score as a cut of point? If so please indicate here

**Response:** We have provided a reference whether it is possible to use mean as cutoff point or not.

When the data analyzed is normally distributed it is possible to use mean as cut off point. Since our data is likert scale data and in addition it is normally distributed, we had used mean as cut off point.

**Comment 4:** There are different logistic regressions please clearly indicate which one is it, am guessing it binary logistic regression so explain clearly.

**Response:** We have added explanation to make it clearer.

**Comment 5:** Determinant and associated factors are different things, please be consistent with associated factors since your design didn’t allow to show cause and effect relationship.

**Response:** The comment is well accepted and corrected accordingly.

***Result section Comments:***

**Comment 1:** How 18,000 is used as cut off point

**Response:** We have used a mean value (18,000) as cut off point. Many published paper were used this cut off points.

**Comment 2:** Is there any observation done by the researchers to confirm the Availability and accessibility of physical activity equipment?

**Response:** The finding didn’t supported by observation. Which is limitation of our study.

**Comment 3:** Can you explain the difference of the effects between western and local movies? You can consider explain why this two variables asked differently on the discussion section if not already stated

**Response:** There is great difference between the two, which follow different dressing, acting etc. styles that motivate, attract the adolescent to follow the same style. So that the adolescent compare their style with them.

**In addition to specific comments, generally we have made to improve the paper to meet high standard of the journal.**
