# Supplementary material for: Self-perception of physical appearance of adolescents and associated factors in Addis Ababa, Ethiopia
Source: PLoS One. 2023 Jan 27;18(1):e0281202. doi: 10.1371/journal.pone.0281202 (PMC9882736; doi:10.1371/journal.pone.0281202)
Supplement: Attachment — Submitted filename: Response to Reviewers and editors comment.docx [file pone.0281202.s006.docx]

**Dear Editor,**

Thank you for giving us the opportunity to submit a revised draft of our manuscript titled (Self-perception of physical appearance of adolescents and associated factors in Addis Ababa, Ethiopia) to *Plos One journal*. We appreciate you and the reviewer for your precious time in reviewing our paper and providing valuable suggestion. It was your valuable and insightful comments that led to possible improvements in the current version. The authors have carefully considered the comments and tried our best to address every one of them. We hope the manuscript after careful revisions meet your high standards. The authors welcome further constructive comments if any.

Below we have provide the point-by-point responses/clarification to editor comments. In addition, all modifications in the manuscript have been highlighted in red in document uploaded named as “Revised Manuscript with Track Changes”.

**Comments from Editor:**

**Comment 1:** Are the confidence intervals "for overall good self-perception of one’s physical appearance was 48.4% [95% CI=48.8, 541]" correct? Please check this

**Response:** It is typing error, and the comment is well accepted and corrected accordingly.

**Comment 2:** Following the discussion, the author needs to mention the limitations of the study. Are there any limitations to this study? The reviewers' comments may assist you in writing these limitations.

**Response:** The comment is well accepted. We included the limitation of the study in the manuscript.

**Comment 3:** Please check the consistency of the font size, for example, the confidence interval in the abstract section

**Response:** Thank you for your valuable comment. The font size was checked throughout the document and corrected as per the journal guideline.

**Comment 4:** Please review your reference list to ensure that it is complete and correct.

**Response:** Checked thoroughly.

**Comment 5:** While revising your submission, please upload your figure files to the Preflight Analysis and Conversion Engine (PACE) digital diagnostic tool

**Response:** Suggestion accepted and uploaded accordingly.
