# Supplementary material for: Self-perception of physical appearance of adolescents and associated factors in Addis Ababa, Ethiopia
Source: PLoS One. 2023 Jan 27;18(1):e0281202. doi: 10.1371/journal.pone.0281202 (PMC9882736; doi:10.1371/journal.pone.0281202)
Supplement: Attachment — Submitted filename: Response to Reviewers and editors comment.docx [file pone.0281202.s007.docx]

**Dear Editor,**

Thank you for giving us the opportunity to submit a revised draft of our manuscript titled (Self-perception of physical appearance of adolescents and associated factors in Addis Ababa, Ethiopia) to *Plos One journal*. We appreciate you and the reviewer for your precious time in reviewing our paper and providing valuable suggestion. It was your valuable and insightful comments that led to possible improvements in the current version. The authors have carefully considered the comments and tried our best to address every one of them. We hope the manuscript after careful revisions meet your high standards. The authors welcome further constructive comments if any.

Below we have provide the point-by-point responses/clarification to editor comments. In addition, all modifications in the manuscript have been highlighted in red in document uploaded named as “Revised Manuscript with Track Changes”.

**Comments from Editor:**

**Comment 1:** In the discussion section line 234, the percentage (48.6%) for good self-perception of one's body appearance is not consistent with the abstract and results. Please also check other inconsistency through results which may delay/affect the acceptance of the manuscript.

**Response:** The correct percentage is 48.4%, it is typing error, and the comment is well accepted and corrected accordingly. Other inconsistence also checked through the document.

**Comment 2:** The results of Table 3 are unclear when it comes to perceptions of dietary and related conditions. How was the percentage calculated for the Likert scale? The analysis section should also describe this. Is column 2 indicating mean (SD)? Which is not clear.

**Response:** The comment well accepted. We included operational definition in the variable definition in the methods and materials section, under study variable sub section. Similarly, we added some explanation under data analysis procedure to make clearer.

**Comment 3:** Researchers used a multistage sampling, which could have higher sampling error than simple random sampling. The study should reported in the limitation.

**Response:** The comment is well accepted, and included in the limitation section.
